# Metal–organic frameworks (MOFs) for arsenic remediation: a brief overview of recent progress

**DOI:** 10.1039/d5ra02420j

**Published:** 2025-06-16

**Authors:** Easar Alam

**Affiliations:** a School of Environmental Engineering, Xuzhou University of Technology Xuzhou 221018 Jiangsu P.R. China easaralam@hotmail.com

## Abstract

Arsenic is one of the most common groundwater contaminants causing serious environmental and health problems worldwide. Arsenic remediation using metal–organic frameworks (MOFs) offers a promising approach for arsenic removal owing to their structural tunability, adjustable pore size, and large surface area. This review explores the adsorption mechanisms, versatile functionality, and dimensionality of MOFs, highlighting their potential for arsenic removal. Various synthesis techniques, arsenic adsorption efficiencies, mechanisms, pH dependencies, adsorption isotherms, and adsorption kinetic models are examined in the context of MOFs used for arsenic removal. Functionalized and hybrid MOFs further improve the adsorption performance and selectivity toward arsenic removal *via* synergetic interactions. The review also discusses the key factors influencing the performance of MOFs, which include pH, competing ions, isotherms, and kinetic models. Despite their advantages, challenges such as hydrolytic stability, scalability, and high cost limit the wide-ranging application of MOFs. However, with advancements in synthesis techniques, structural modifications and integration into practical water treatment systems, MOFs can provide a sustainable and large-scale solution for arsenic removal. This review provides an overview of the recent progress of MOFs in the field of arsenic remediation and suggests some future directions for their further improvement in practical applicability under real-world conditions.

## Abstract 2

Arsenic is one of the most common groundwater contaminants causing serious environmental and health problems worldwide.

## Graphical abstract

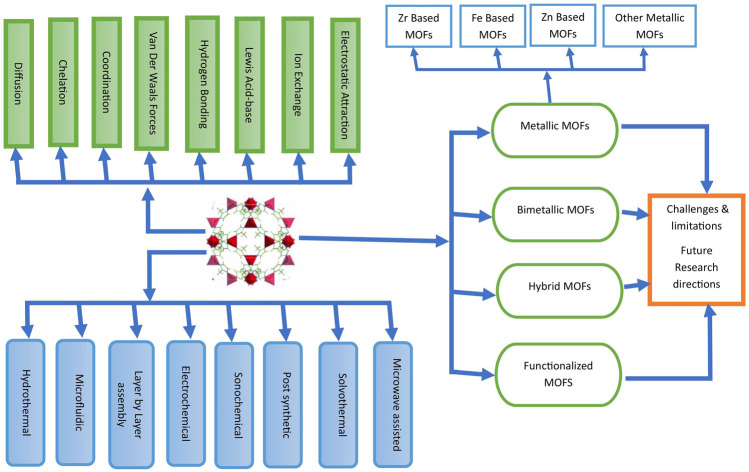

## 1 Introduction

Water pollution has threatened the entire ecosystem and humans, thereby affecting billions of people around the world. Various contaminants such as chemicals, heavy metals, pathogens, and nutrients from various sources such as industrial discharges, agricultural runoff and municipal wastes contribute to the pollution of water systems such as rivers, lakes, and seas.^1,2^ Industrial processing activities such as mining, manufacturing or energy generation release hazardous byproducts such as heavy metals into the water systems.^3^ This contamination diminishes aquatic life, alters food webs and causes loss of biodiversity over time, which have significant impacts on aquatic biodiversity and human populations.^4^ One of the most well-known toxicants that emerge from water supply and has harmed millions of people around the globe is arsenic. Waterborne arsenic originates from several natural and manmade processes.^5^ Anthropogenic causes of water pollution include coal-consuming power plants, mining operations and the utilization of arsenic-containing manures and pesticides. These activities increase arsenic pollution through direct emission or runoff to water bodies.^6,7^

In water, arsenic mainly exists in two oxidation states: As^3+^ and As^5+^. Both are highly toxic and pose serious health hazards. Long-term exposure to As^3+^ in drinking water can cause life-threatening chronic diseases such as malignancies and cardiovascular disease.^8^ As^3+^ is 60 times more toxic than As^5+^ in drinking water and is one of the most toxic pollutants present in our surroundings and the environment.^9^ Arsenic is well known to cause long-term toxicity that has severe health consequences, including malignancies, such as lung cancer, bladder cancer, skin cancer; cardiovascular diseases, and diabetes.^10^ The health effects are even more severe in places where the population depends on arsenic-polluted groundwater for drinking and crop irrigation. The environmental consequences of arsenic contamination are equally profound. Both the degradation of aquatic ecosystems and mortalities of aquatic organisms are triggered by arsenic-contaminated water systems.^11,12^

To mitigate the harmful effects of arsenic pollution on human health and environment, effective methods for arsenic removal are necessary. Several methods, including chemical precipitation, ion exchange, adsorption, membrane filtration, and biological treatments, are used to remove arsenic from contaminated water.^13^ Each of these approaches has its own advantages and disadvantages. Coagulation is a low-cost, widely used method that removes particulate and colloidal arsenic by clumping particles into easily removable flocs. However, it is largely ineffective against dissolved arsenic, particularly As(iii).^14^ Precipitation removes arsenic by converting it into insoluble compounds that can be separated through filtration or sedimentation, effectively reducing arsenic levels when optimized;^15^ however, it generates toxic sludge with low metal concentration that requires careful disposal.^16^ Membrane filtration techniques such as nanofiltration and reverse osmosis provide high rejection rates for arsenic and other contaminants but involve high energy demands, potential membrane fouling, and expensive maintenance, making it less viable for decentralized or low-resource settings.^17–19^ Ion exchange processes offer good selectivity for certain arsenic species, allowing for effective removal from aqueous solutions; however, their performance is often limited by fouling caused by various substances, competition with coexisting ions, and the need for periodic chemical treatment to maintain function and regenerate the resins.^20–22^ Biological treatment for arsenic removal processes such as the use of iron-oxidizing bacteria offer a cost-effective and environmentally friendly option for detoxifying arsenic in contaminated water,^23^ but challenges including the varying oxidation states of arsenic persist, which can complicate the removal efficiency.^24^ Adsorption is one of the most practical and extensively applied strategies for arsenic removal due to its low cost, simplicity, and versatility. Different adsorbents such as activated carbons, iron oxides, polymers, graphenes, raw materials, dopants, and biosorbents are used for arsenic removal. These adsorbents have shown different removal efficiencies of arsenic under different conditions.^25,26^ Furthermore, the mechanisms of adsorption processes are easy to operate and require little maintenance.^27,28^

Among different adsorption-related techniques, those integrating advanced materials such as metal–organic frameworks (MOFs) have gained increasing attention owing to their efficiency in arsenic removal. Metal–organic frameworks (MOFs) are a unique class of materials composed of metal ions or clusters coordinated to organic linkers. This unique architecture grants them tunable micro/macro properties and a crystalline porous structure with extraordinarily high surface areas and controllable pore sizes.^29–31^ By carefully choosing metal components and organic ligands, researchers harness the synthetic versatility of MOFs to develop highly effective adsorbents for water purification. Such materials displayed high adsorption efficiency, tunable porosity, and adjustable chemical compositions to specifically target contaminants including arsenic.^32–34^ MOFs with tunable structures, high specific surface areas, and selective ion-capture capabilities demonstrate strong performance in arsenic extraction; however, their performance is influenced by pH and framework composition.^35–37^ Tunability facilitates the precise synthesis of metal nodes and linkers to enhance interactions with arsenic species, which can be further improved through functional groups to increase the effectiveness of adsorption.^38,39^ For practical applications, the aqueous stability of MOFs is crucial for their effective use in water treatment. To keep the structural integrity during adsorption, it is important to maintain their shape, like crystals, especially when the pH levels are controlled.^39^ Therefore, a thorough study is required to discuss the latest developments in MOF materials for arsenic removal. Although there are few review studies on the application of MOFs such as hybrid MOF composites,^40^ organic arsenic adsorption,^41^ Fe-based MOFs,^42^ tuned porous MOFs and COFs,^43^ and aqueous arsenic by MOFs^44^ in arsenic remediation, there is a significant gap in the literature concerning comprehensions and up-to-date analysis focusing on arsenic removal using different MOFs. Most of these available reviews are either limited in scope or need to be updated. Hence, a systematic review is essential to evaluate the recent advances, identify current trends and novel approaches, and address key challenges and future research directions.

The aim of this study is to present a systematic review of the current available literature on the performance and mechanisms of action of different metal–organic frameworks (MOFs) used for arsenic treatment. This study creates a single platform to provide scientists, engineers and environmentalists with resources to work together to combat water pollution by systematically tracking and reporting relevant developments in the field. Such findings could also help enable sustainable arsenic removal technologies in water systems to be developed.

## 2 Overview of MOFs

### 2.1 Structure and properties

#### 2.1.1 Surface area

MOFs are known for their high specific surface areas and good adsorption capacities. MOFs have a higher surface area than that of the conventional substances, making them more effective in removing pollutants from various environments.^45^ MOFs with higher surface areas showcase enhanced arsenic adsorption potential, mainly due to the increased availability of active sites that facilitate interactions among the adsorbent and the arsenic species. Gly@UiO-66(Zr), characterized by a specific surface area of 582 m^3^ g^−1^, effectively removed 301.4 mg g^−1^ of arsenic(iii) from water.^46^ Furthermore, the pore sizes of MOFs enable the design of materials specifically optimized for selective adsorption of arsenic ions, tailored to match their length and shape.^36^

#### 2.1.2 Tunability

The tunable nature of MOFs, enabled through ligand functionalization, metal node selection, and composite designs, makes them exceedingly effective for arsenic removal from water. Ligand functionalization, including the incorporation of thiol (–SH) groups, considerably enhances arsenic affinity. Thiol-modified UiO-66-SH achieved an adsorption capacity of 53.31 mg g^−1^ of As(v) at pH 3, due to the strong thiol-arsenic interaction.^47^ Metallic node selection also plays a critical role in modulating arsenic uptake. Fe-based MOFs such as Fe_3_O_4_@MIL-101(Cr) leverage the strong affinity between Fe and AsO_4_^3−^ ions, attaining an impressive As(iii) removal capacity of 121 mg g^−1^,^48^ while Zr-based frameworks such as UiO-66 used Zr–O–As chelation to attain an adsorption capacity up to 200 mg g^−1^ at pH 7.^39^ Furthermore, MOF composite designs, consisting of MOF/graphene hybrids, combine the structural advantages of MOFs with the electrical conductivity of graphenes, enhancing both adsorption and electrochemical detection of arsenic.^49^ These strategies collectively highlight the flexibility and adaptability of MOFs for green arsenic remediation.

#### 2.1.3 Chemical stability

The chemical stability of MOFs is an important factor determining their effectiveness for arsenic elimination from water. MOFs must maintain their structural integrity under aqueous and variable pH conditions to function reliably in different environmental applications. An indium-based MOF maintained its structure at different pH levels, supporting its suitability for arsenic remediation.^50^ It was emphasized that the stability of MOFs under practical conditions is critical for maintaining high adsorption efficiency.^35^ Zinc-based MOF composites exhibit remarkable stability and high arsenic elimination ability (99%), highlighting the critical role of material design in influencing both durability and efficacy.^51^ Furthermore, the structural stability and the presence of active sites in defective Zr-MOFs significantly enhanced arsenic adsorption (301.4 mg g^−1^ at pH = 8).^46^

#### 2.1.4 Functional diversity

The incorporation of functional groups into the MOF structure is highly beneficial, as these functional groups can improve their adsorption capacity. Interactions among functional groups and target molecules (hydrogen bonding or π–π stacking) can effectively increase the adsorption efficiency.^52^ For instance, amino-functionalized MIL-68(Al) achieved a notable improvement in As(v) removal efficiency of 99.87% as compared to 74.4% for its unmodified counterpart, due to the increased electron-rich sites and enhanced hydrogen bonding capabilities.^53^ Furthermore, Ce-MIL-101-NH_2_ has proved good adsorption capacities for both phosphate (341.5 mg g^−1^) and As(v) (249 mg g^−1^). The material's effectiveness was attributed to electrostatic attraction and complexation among Fe/Ce sites and oxyanions, displaying exquisite and selective performance even in the presence of competing anions.^54^

#### 2.1.5 Crystallinity

The crystallinity of MOFs extensively affects their efficacy in arsenic elimination from polluted water. MOFs are characterized by their well-ordered crystalline structures, which facilitate efficient adsorption capacities.^55^ High crystallinity is vital for ensuring that the adsorbent keeps its structural integrity and binding sites during interaction with adsorbates including arsenic. The high crystallinity allows for improved stability under operational conditions, which is vital for maintaining their performance over time.^56^ Zirconium-based MOFs like UiO-66 exhibited brilliant stability in aqueous environments, combining high adsorption ability for arsenic(v) with structural integrity.^57^ Such stability also aids in maintaining their functional properties during the adsorption of contaminants such as arsenate ions.^58^ Moreover, the presence of functional binding sites inside the framework, dictated by its crystalline structure, contributed for the selective adsorption of arsenic. The specific coordination interactions between arsenic species and functional groups in the MOFs are optimized in a well-crystallized framework, fostering improved binding affinities.^59,60^

#### 2.1.6 Chemical stability

**Fig. 1:**
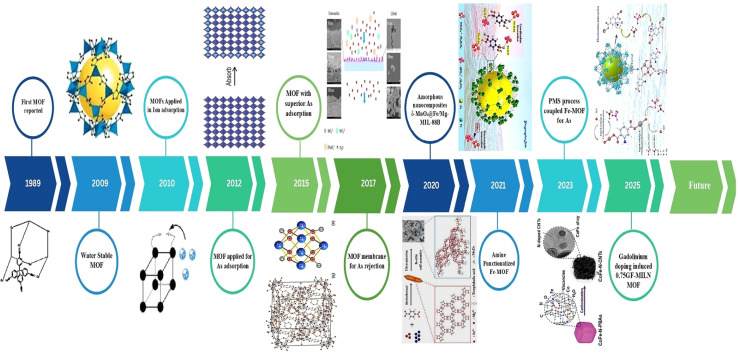
Timeline of the development of MOFs for arsenic removal (1989,^65^ 2009,^66^ 2010,^67^ 2012,^68^ 2015,^69^ 2017,^70^ 2020,^71^ 2023,^72^ and 2025 (ref. 73)).

The chemical stability of MOFs under diverse environmental conditions is vital for effective arsenic removal. Recent research highlights the need for improved resistance to water and harsh environments to ensure reliable performance in water treatment.^61^ The chemical stability of MOFs directly correlated with the strength of metal–ligand bonds they possess.^62^ The inherent stability of MOFs is crucial because arsenic exists in different oxidation states, mainly As(iii) and As(v), each with distinctive solubility and reactivity properties. For example, iron-based MOFs (Fe/Mg-MIL-88B) exhibited high adsorption potential (*i.e.* 303.6 mg g^−1^) for As(v) attributed to their high surface area, tunable pore size, and strong chemical stability.^63^ While MOFs are recognized for their chemical stability and efficacy in arsenic removal, challenges such as contaminant diversity and fluctuating water chemistry still persist. Ongoing research focuses on modifying MOFs, such as by incorporating various metallic ions to enhance both balance and adsorption ability, demonstrating a synergistic technique to improve its performance.^64^ A timeline for the development of MOFs for arsenic removal is illustrated in Fig. 1.

### 2.2 Synthesis techniques of MOFs

Different synthetic strategies have been used to prepare MOFs, each of which has certain advantages and corresponding applications in important fields. The most commonly used synthesis methods for MOFs are as follows.

#### 2.2.1 Hydrothermal and solvothermal method

Hydrothermal method is one of the simplest routes for MOF synthesis, in which metal salts and organic ligands are dissolved in a solvent and heated in a sealed vessel to promote crystallization.^74^ The hydrothermal approach facilitates the growth of MOFs under extended temperatures and pressures.^75^ Due to steric and scale limitations in MOF synthesis, this technique offers clear advantages by means of producing highly crystalline materials, as the conditions enhance reactant solubility and improved crystallization kinetics.^76^ One prominent example of MOFs synthesized through hydrothermal methods is UiO-66, a zirconium-based framework that has proven notable efficacy for arsenic adsorption. UiO-66 can efficaciously capture arsenate ions from water, with a tremendous adsorption capacity up to 303 mg g^−1^ under certain conditions.^69^ Moreover, Zn-based MOFs such as hybrid Fe_3_O_4_@ZIF-8 composites synthesized *via* a hydrothermal route exhibit promising results in arsenic elimination. This composite effectively integrates magnetic properties with the inherent adsorption abilities of ZIF-8, facilitating the ease of recovery after treatment, a vital factor for wastewater applications.^77^ Furthermore, hydrothermal synthesis is considered as environmentally friendly, because it solely relies on water, avoiding the use of hazardous natural solvents.^78^

The solvothermal method is similar to the hydrothermal method, except that it uses organic solvents such as DMF (dimethylformamide) or DMSO (dimethyl-sulfoxide), instead of water in the synthetic procedure.^74^ This strategy provides hierarchical control over reaction parameters, which may result in MOFs with new topologies and morphologies.^79^ Both hydrothermal and solvothermal techniques offer enormous benefits, which include high yield and purity.^80^ Parameters such as temperature, strain, and solvent composition may be precisely adjusted to control the dimensions and shape of MOFs at the atomic level, which is important for optimizing their performance.^81^ Hydrothermal synthesis, which uses water as the solvent under increased temperature and pressure, often yields MOFs with better crystallinity and better output than solvothermal techniques. Studies have demonstrated the effectiveness of solvothermally synthesized MOFs for arsenic elimination, for example, Ce-MOFs synthesized solvothermally exhibited effective removal of arsenic along with other contaminants.^37^

#### 2.2.2 Microwave-assisted synthesis

Microwave-assisted synthesis is a rapid and efficient technique for the preparation of MOFs, utilizing microwave irradiation to accelerate the reaction kinetics and enhance the material quality.^82^ By empowering speedy and uniform heating of the reaction mixture, this method notably reduces synthesis time, frequently producing phase-pure MOFs within 10 min in comparison to hours or days required for conventional hydrothermal or solvothermal methods.^83^ This synthesis regularly results in better yields and improved reproducibility of MOF materials due to its controlled environment.^84^ The tremendous properties of microwave-synthesized MOFs, including extended porosity and surface area, are vital for effective adsorption process, especially in removing hazardous contaminants.^85^ The continuous flow of microwave-assisted reactors can facilitate the synthesis of nanomaterials with controlled size and morphology, thus optimizing their overall performance in specific applications including metal ion capture.^86^ MOF-808 synthesized by the microwave-assisted method exhibit efficient arsenic removal. The results suggested that MOFs achieved significant adsorption of arsenic from aqueous environments, thereby facilitating treatment strategies that could be applied in other water sources.^87^ Moreover, advances in microwave-assisted synthesis have facilitated process optimization and scale-up, paving the way for commercial adoption in water purification technologies.^88^

#### 2.2.3 Microfluidic synthesis

Microfluidic synthesis is considered to be a powerful state-of-the-art tool for MOF production that outperforms conventional methods in many aspects. Using microfluidic devices, MOF syntheses have been performed *via* a continuous-flow reaction, which makes it possible to combine reactants in real time, achieving very fast yet uniform synthesis of MOFs with intimate control over morphology and properties. Microfluidic synthesis offers high mixing efficiency, which translates into improved reaction kinetics and reduced batch-to-batch variability.^89,90^ Micromixing and mass transfer in microfluidic reactors occur rapidly, which increased the reaction rates and allowed the synthesis of catalyst-decorated conductive MOF thin films.^91^ Moreover, by designing microfluidic systems to produce certain flow patterns, it is possible to improve both mixing and reaction conditions.^92^ A reported microfluidic method allowed the continuous synthesis of MOF crystals, yielding post-synthesis high-quality materials for a wide range of applications.^93^ Another important area is the use of microfluidics along with other technologies for the formation of hybrid materials, advanced catalysts, and aerosol-assisted synthesis.^94,95^ It is proved that in the microfluidic method, the droplet allows reactions to occur in confined volumes, resulting in highly effective heat and mass transfer that significantly accelerates the synthesis process compared to the traditional bulk methods.^95^

#### 2.2.4 Electrochemical synthesis

Electrochemical synthesis of MOFs has attracted sizeable interest because of its mild conditions, rapid processing, and low energy requirements in comparison to conventional strategies like solvothermal synthesis. The strategies which include cathodic and anodic deposition offer specific control over response parameters and materials properties. For instance, cathodic deposition of MOFs like Cu-BTC allows uniform film formation under mild conditions, making it suitable for different applications.^96,97^ One key benefit of electrochemical strategies is the feasibility of synthesis at room temperature and under atmospheric conditions, avoiding the need for extreme temperatures and harsh solvents.^98^ This is important in minimizing the environmental effect related to the synthesis of these materials.^99^ The direct electrochemical method, particularly the anodic dissolution of metal sources, generates metal ions that effectively react with organic linkers to form planar and intricate 2D and 3D MOF structures.^100^ Moreover, the precise manipulation of reaction conditions which include electrolyte composition and current density can lead to intricate control over the structural properties of the MOFs produced.^101^

The applications of electrochemically synthesized MOFs are vast and increasing. One of the most prominent areas of application is electrochemical sensing. For example, Cu-based MOFs have shown promise in the non-enzymatic detection of glucose because of their advanced electrochemical interest.^102^ Similarly, the unique features of various MOFs have facilitated their use in catalysis, along with the evolution of hydrogen from water.^103^ MOFs synthesized *via* electrochemical routes have additionally been employed in the synthesis of nanocomposites that decorate electric-powered conductor materials, important for application in supercapacitors and energy storage systems.^104^ Latest advances have seen the development of composite materials, wherein MOFs are integrated with conductive polymers or nanoparticles, improving their electrochemical performance.^105^ Such hybrids influence the high surface area and porosity of MOFs while addressing problems associated with their inherent electric insulating nature.^104^ The effective integration of various materials has consequently broadened the functional possibilities of MOFs in environmental sensing and electricity storage programs.^106^

#### 2.2.5 Layer-by-layer (LbL) assembly

The layer-by-layer (LbL) assembly is a precise method for MOF fabrication, where metal ions and organic linkers are sequentially deposited onto a substrate to form well-ordered crystalline films.^107^ The LbL process is reported to be compatible to generate crystalline heterolayers by adding different types of MOFs to each other to achieve better film functional properties.^108^ One of the key benefits of the LbL method is that it allows precise control over MOF film thickness by adjusting the cycle number, when the structural properties can be tailored by modifying the concentration at deposition.^109^ Such control is essential for optimizing the MOF performance across various applications, as it directly influences their structural and functional properties.^110^ Layer-by-layer approach offers a promising route for directly incorporating guest species into the MOF lattice, significantly expanding the scope for functionalizing these materials and tailoring their properties for specific applications.^111^ Moreover, the functional versatility of the LbL method allows it to be seamlessly integrated with liquid-phase epitaxy and other techniques, enabling scalable fabrication of complex multilayer MOF architectures.^112^ Recent advancements in this field have focused on enhancing the efficiency of the LbL process, enabling the rapid synthesis of high-quality MOF films within significantly reduced timescale.^113^

**Fig. 2:**
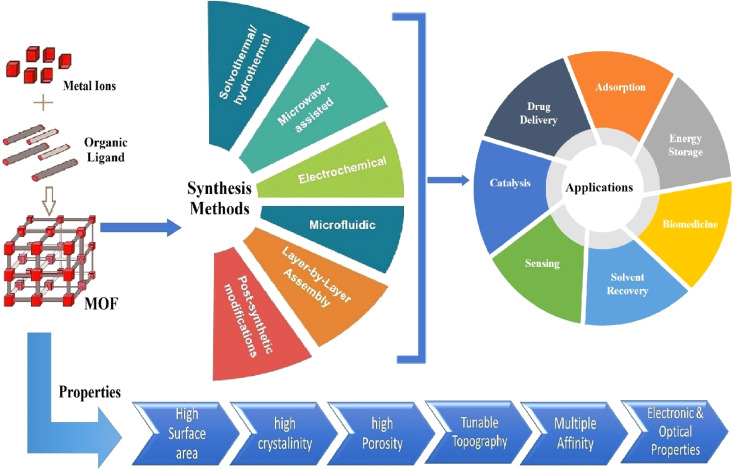
Synthesis routes and applications of MOFs.

Some of the most commonly used synthesis routes and the applications of MOFs for arsenic removal are illustrated in Fig. 2.

#### 2.2.6 Post-synthetic modifications

One of the key advantages of MOFs is their ability to undergo post-synthetic modification (PSM), which enables the fine-tuning of their properties after initial synthesis. This strategy allows the introduction of functional groups or guest molecules into the framework, modifying both metal and organic sites to customize MOFs for specific applications including adsorption, gas storage, catalysis, and sensing.^114,115^ A widely used PSM approach involves ligand exchange, where pre-existing ligands within MOFs are replaced with new ones to impart different functionalities and stability.^116^ Additionally, metal ion exchange can incorporate different metal ions into the framework, allowing the fine tuning of electronic and catalytic properties to develop highly active MOFs.^117^ Covalent PSM further enables precise property adjustments; for example, modifying a spin-crossover MOFs can induce a single-crystal-to-single-crystal transformation, altering spin-switching temperature and cooperativity.^118^ Another innovative method involves hydrolytic PSM to convert microporous MOFs into hierarchical micro and mesoporous structures, enhancing their utility.^119^

The adsorption efficiency of MOFs for arsenic depends heavily on their metal nodes and organic linkers. Studies demonstrate that iron-trimesate porous solids effectively adsorb arsenate at neutral pH due to their structural versatility and PSM capabilities.^120^ Similarly, Ce(iv)-based metal–organic gels exhibited improved arsenic adsorption *via* structural modifications that increased active sites and reduced diffusion limitations.^121^ Introducing functional groups *via* PSM can significantly enhance the sorption properties of amine-modified UiO-67, which showed markedly better arsenic adsorption than its unmodified counterpart.^122^ Manganese-doped defective UiO-66 further improved As(iii) removal efficiency, highlighting the potential of defect engineering in MOF design.^123^

PSM also broadens the functional group scope within MOFs, enhancing their ability to capture toxic compounds like arsenates.^124^ Ionic MOFs can adapt their structures for selective arsenic adsorption,^125^ while integrating MOFs into polymer matrices yields nanofibrous composites with improved arsenic-removal capabilities.^51^ Modifications in Zr-based MOFs enhanced hydrophilicity and chemical stability, and improved arsenate binding efficiency under different pH and redox conditions.^39,59^ Click chemistry offers another efficient PSM route, allowing diverse functional groups to be attached while maintaining the MOF integrity.^116^ Such strategies enable the development of multifunctional MOFs suitable for environmental remediation.^126^ The modular design of MOFs, combined with PSM, creates chemically heterogeneous environments ideal for selective adsorption or catalysis.^115,127^ Moreover, nanoparticle encapsulation *via* PSM enhances the catalytic performance and stability,^114,128^ underscoring the immense potential of these tunable composites in arsenic removal and other applications. A brief comparison of different synthesis methods for MOFs is presented in Table 1.

**Table 1:** Comparison of different synthesis methods for MOF synthesis

Synthesis method	Description	Advantages	Disadvantages	Ref. #
Solvothermal/hydrothermal	Heats metal salts and organic linkers in a sealed jar after dissolving them in a solvent	• One-step synthesis	• Long reaction times	22 and 129
		• Produce single crystals	• Requires large amounts of solvent	
		• Moderate temperature conditions	• Potential for unwanted by-products	
Microwave-assisted	Utilizes microwave radiation to rapidly heat the reaction mixture	• Rapid synthesis (mins)	• Limited scalability	22 and 129
		• High purity products	• Requires specialized equipment	
		• Uniform morphology		
		• Eco-friendly		
Mechanochemical	Involves grinding solid reactants together, often using ball milling	• Solvent-free (green chemistry)	• Products may be amorphous, hindering structural analysis	22 and 129
		• Short reaction times	• Limited control over particle size	
		• High efficiency		
Electrochemical	Metal ions are generated electrochemically and react with organic linkers in solution	• Mild reaction conditions	• Limited to conductive metal precursors	22 and 129
		• Rapid synthesis	• Requires specialized equipment	
		• Avoids hazardous solvents		
Sonochemical	Employs ultrasonic waves to induce cavitation, promoting chemical reactions	• Fast reaction times	• Limited control over crystal growth	22 and 129
		• Improved dispersion of reactants	• Challenges in scaling up	
Spray drying	Atomizes a precursor solution into fine droplets, which are rapidly dried to form MOF particles	• Continuous and scalable process	• Potential loss of crystallinity	22 and 129
		• Suitable for industrial applications	• Requires optimization for each MOF	
Template-assisted	Uses a pre-existing structure (*e.g.*, polymer or silica) to guide MOF formation	• Precise control over morphology	• Additional steps to remove templates	129 and 130
		• Enables hierarchical structures	• Potential environmental concerns with template materials	

## 3 Mechanisms and factors affecting MOFs

### 3.1 Adsorption mechanisms

Adsorption is a process through which ions, atoms, or molecules (adsorbates) attach themselves to the surface of a solid or a liquid (adsorbent). Some of the most important factors that affect adsorption are the properties of the adsorbent and adsorbate, temperature, pressure, and surface area of the adsorbent.^131,132^ The mechanisms of MOFs that interact with arsenic species are mainly described as electrostatic attraction, ion exchange, and chelation.

Electrostatic attraction dominates the adsorption behavior of arsenic species onto MOFs. Many MOFs, in particular, are charged at their surfaces and could preferentially interact with the oppositely charged arsenic species. The Zr-based MOF, UiO-66, has an excellent ability to eliminate arsenic from water due to its highly porous nature and positively charged sites in the crystal framework attractive to negatively charged arsenate ions.^69^ Similarly, the results indicated that arsenate adsorption in RT-Zn-MOF-74 occurs through electrostatic interactions at certain pH intervals, where the conditions are conducive to electrostatic interactions.^38^ The electrostatic forces are significant for the stabilization of MOF structures and the entrapment of anionic species, effectively improving the adsorption capacity.^133^

Another important ion-adsorptive mechanism of MOFs involves interactions with arsenic species beyond simple absorption. The presence of some functional groups of the MOFs could facilitate ion transfer between the MOF framework and the arsenic species present in the solution. The significant accumulation of arsenic on UiO-66 was confirmed by a study, suggesting that counter ions could exchange and interact directly with the MOF framework.^39^ Additionally, the ion exchange capacity of dihydrotetrazine-functionalized Zr-MOFs exhibited a higher exchange capacity than that of unmodified MOFs, confirming that the functionalization of MOFs could further enhance their potential to entrap arsenate ions.^57^

Chelation is one of the most extensively studied interaction mechanisms for the removal of arsenic by MOFs. Some MOFs can significantly enhance their arsenic uptake capacity by stable complexation with arsenic species. For example, UiO-66 has demonstrated efficient chelation of both As(iii) and As(v).^134^ The integration of these distinct mechanisms allows MOFs to overcome current state-of-the-art approaches for arsenic removal from polluted water, positioning MOFs as promising candidates for environmental remediation.

### 3.2 Functional groups and surface chemistry

Various strategies such as functionalization, surface pegylation, and composite materials have been proposed in the literature to enhance the selective adsorption of target molecules by modifying the structural and chemical properties of MOFs. Organic linkers usually contain various functional groups that considerably affect the chemical behavior, stability, and application options of the MOFs. Carboxylate groups are among the most important functional groups in MOFs and commonly found in linkers such as terephthalic acid. The carboxylate portion coordinates to metal nodes through oxygen atoms, forming strong and stable frameworks. This coordination not only ensures the structural integrity of MOFs, but also enhances the ability to adsorb gases and other molecules.^135,136^ Another interesting functional group in MOFs is the amino group, which significantly enhances the basicity of the framework. The amino group allows diverse interaction modes between the MOFs and different organic pollutants, broadening their potential applications in environmental remediation.^137,138^

The addition of hydroxyl groups to MOFs significantly increases hydrophilicity, leading to strong hydrogen bonding with water and other polar solvents. The –OH groups not only participate in hydrogen bonding, but also increase in numbers too, enhancing the stability of MOFs in water-based environments. Additionally, hydroxyl groups can act as active sites for further chemical modifications or host–guest interactions, thereby promoting the functionality of the MOFs.^139,140^ Researchers introduced an acidic functionality into MOFs by incorporating sulfonic acid groups, making them suitable for catalysis. The acidity of the –SO_3_H groups enhances catalytic activity by participating in different reactions. Moreover, sulfonic acids groups promoted the adsorption of more polar molecules, expanding the range of potential applications of these materials.^141,142^ Some functional groups such as halides, thiols, and azides offer a diverse reactivity for post-synthetic modifications (PSM) or selective interactions. Halides can facilitate coordination to metal ions, and thiols can provide covalent bonding sites for other molecules, increasing the functionality of the MOFs. This is an attractive feature of azide groups as they are one of the most popular click chemistry partners, allowing the integration of many functionalities onto the MOF surface.^35,143^

Surface chemistry of MOFs also plays a crucial role in their function and application. Organic linkers impart hydrophilicity or hydrophobicity characteristics of MOFs based on their functional groups, whereas hydroxyl (–OH) groups increase affinity for water and methyl (–CH_3_) groups increase hydrophobicity.^139,144^ The presence of open metal active sites in MOF structures enables these entities to function as adsorption sites, therefore increasing their capability for different chemical processes and separations.^139^ Using post-synthetic modification (PSM) techniques including click chemistry, the surface chemistry of MOFs can be modified in order to tailor them for certain applications without compromising their structural integrity.^145^ This design adaptability is particularly beneficial for improving molecule-target interactions, evidenced by functionalized MOFs exhibiting improved binding affinities for proteins and other analytes.^141^ Examples include organic linkers, metal nodes, or surface modifications, which render MOFs versatile materials for a wide range of advanced applications, ranging from catalysis to biomedical applications.^146^

The introduction of amino and mercapto functional groups into the internal structure of a Zn(ii)-imidazole framework improved the adsorbent capacity (*i.e.* 718 mg g^−1^) of As(v).^133^ These changes create vast chemical spots for bonding, attuned by electrostatic interactions, thereby significantly improving the efficiency of arsenic grafting. Another useful strategy for the stability and adsorption capacity of arsenic by MOFs is surface pegylation. Pegylation complements the relationship among arsenic ions and the MOF surface, improving both adsorption capacity and selectivity for arsenate whilst also enhancing stability against leaching and degradation in complicated wastewater matrices.^38^

### 3.3 pH and other environmental factors

The impact of pH on adsorption is an essential component that influences the efficacy of diverse adsorbents in eliminating pollutants from aqueous solutions. The changes in solution's pH modify both the surface charge of the adsorbent and the ionization state of the adsorbate.^147^ The pH of solution is a very crucial factor that affects the arsenic adsorption capacity of MOFs.^35^ The UiO-66 framework showed a desirable adsorptive capacity (303 mg g^−1^) at pH 2.^69^ At lower pH levels, the adsorption of arsenic species, particularly As(v), is significantly increased, due to the protonation of the MOF surface, which results in a positively charged surface. This promotes strong electrostatic attraction with negatively charged arsenate ions, facilitating effective adsorption. On the contrary, at higher pH levels, the adsorption efficiency is often lower because hydroxide ions can compete with adsorbates by occupying the active sites on the MOF surface.^13^

Temperature is also crucial for the adsorption of arsenic. Thermodynamic parameters revealed that arsenic adsorption was spontaneous and endothermic at a temperature from 296 to 332 K.^148^ It shows that the mobility of arsenic ions and kinetic energy for the adsorption process will be enhanced at higher temperatures, thus facilitating the interaction between arsenic and MOFs. As the MOF temperature increases, their maximum adsorption capacities toward arsenic also increase, suggesting a more thermodynamically favorable sorption type.^35^

The efficiency of arsenic adsorption onto MOFs can be severely affected by the competing ions in the solution. In particular, the presence of phosphate ions was reported to compete with arsenate for active sites on a MOF surface and thus inhibit arsenic adsorption.^53^ Due to the similar chemistry between arsenate and phosphate, this competition occurs, allowing phosphates to antagonize the overall capacity for arsenic adsorption.^149^ In another study based on the MOF-74 framework, increased concentrations of phosphate ions considerably reduced the efficiency of arsenic adsorption.^37^

### 3.4 Physical and chemical adsorption

Physisorption refers to the weak van der Waals interactions between the adsorbate and the adsorbent. This is known as physical adsorption, which is reversible and has energy changes that are lower than chemical adsorption.^150^ Chemisorption involves the formation of a strong chemical bond between the adsorbate and the adsorbent, accompanied by a significant energy change. This process usually requires a higher activation energy, and is generally irreversible.^151^ A primary mechanism of arsenic adsorption in MOFs is chemisorption, which involves the formation of strong chemical bonds between arsenic species and active sites on the MOFs. Zr-MOFs, like UiO-66, exhibit a high capacity for arsenate uptake, with a wide surface area and a large number of active sites on the surface.^39^ Amino functional groups introduce preferential adsorption sites within imidazole frameworks for arsenic, lead and Hg ions primary through noncovalent interactions.^133^ Bimetallic framework demonstrated improved regeneration and selectivity, optimizing the adsorption performance for arsenic removal. This represents a considerable progress with respect to both capacity and selectivity compared to conventional adsorbents.^63^ The redox mechanism involves the redox-active functional groups in MOFs to actively immobilize and convert arsenic to other chemical states *via* an electrochemical process.^152^

### 3.5 Metal–ligand interactions

Metal–ligand exchanges between MOFs and arsenic, predominantly the connection of arsenate species to metal nodes, play a key role in governing adsorption mechanisms and determining the efficiency of arsenic removal from contaminated water.

The open metal sites on MOFs interact with arsenic species, enabling their binding through coordination chemistry. Zr-based MOFs such as UIO-66 exhibited high adsorption capacity for As(v), due to the presence of exposed metal ions. These sites facilitate strong Lewis's acid–based interactions with oxyanions, leading to the formation of inner-sphere coordination complexes with arsenate, resulting in a significant increase in adsorptive capacity.^69^ The available open sites efficiently capture both As(iii) and As(v), however, competing phosphate ions may form stronger interactions with the metal centers. The interaction strength is further increased by the tetrahedral geometry of As(v), which closely aligns with the coordination geometry of metal sites, leading to more stable binding.^77^

The selectivity and adsorption capacity of the MOFs for arsenic species can vary significantly based on the functionalization of ligands. The adsorption characteristics can be tailored by placing certain functional groups in the ligand framework for better interaction of arsenic. The insertion of amino (NH_2_) or hydroxyl (–OH) groups presents additional hydrogen bonding locations and greatly improves the efficient capture rate for As(iii) or As(v).^153^ The amino functionalization of the MIL-68 (Al) MOFs resulted in a great increase in arsenate removal. This enhancement is credited to the increase in the number of electron-rich nitrogen sites and positive charge introduced by the amine groups, which strengthens hydrogen bonds and enhanced the adsorption kinetics.^53^ This modification of MOFs to add functional groups is critical because it improves selectivity by strengthening the interaction between the oxyanions of these ions (arsenate) and adsorbent through hydrogen bonds and electrostatic interaction.^69^ These amine side groups not only increased the binding affinity but also enhanced the selectivity for binding of arsenic around other anion species, which was a major competing analyte in the solution.^153^

**Fig. 3:**
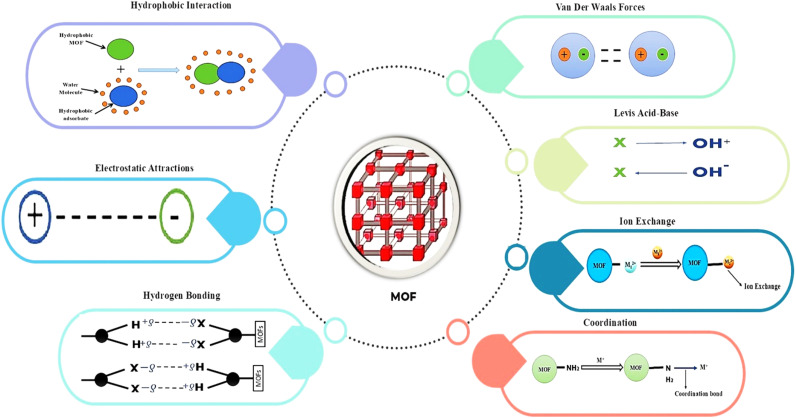
Different adsorption mechanisms and interactions of MOFs with arsenic ions.

Hydrogen bonding plays an important role in the adsorption of arsenic through MOFs. There are hydroxyl groups on the surface of MOFs, which could be conducive to the formation of hydrogen bonds with arsenic species, especially As(iii).^77^ The hydroxyl groups present in the structure provided a complexing site for arsenic species, which increased the total amount of adsorbed arsenic from the aqueous solution. The ability of MOFs to interact with arsenic species through hydrogen bonds facilitated the adsorption process, while also helping the adsorbed species to be stable against desorption under fluctuating environmental conditions.^69^ Some of the most commonly used adsorption mechanisms and factors affecting MOFs in arsenic removal are shown in Fig. 3.

## 4 Different MOFs used for arsenic removal

Adsorption is a well-known method for the treatment of different types of wastewater pollutants. It is commonly used to eliminate heavy metals, such as arsenic from water, based on the interactions between various arsenic species and the surface of adsorbents.^25^ This process is affected by some factors including temperature, pressure, surface area, pH, and the chemical properties of adsorbents and adsorbates.^154^ Adsorption is strongly influenced by pH and plays an important factor in the ability of adsorbents to treat pollutants from water.^147^*Q*_max_ refers to the theoretical maximum adsorbate load of an adsorbent based on conditions that are present for a given system, and *Q*_e_ is the amount of adsorbate remaining on the adsorbent after the system reacts to reach equilibrated conditions.^155^ Typically, *Q*_max_ was calculated according to isotherm models such as the Langmuir model. This model assumes monolayer adsorption on a surface with a fixed number of identical sites.^156^ Different parameters such as surface area, pore structure of adsorbent and micro-fracture characteristics of the adsorbent and adsorbate influence *Q*_max_. Higher adsorption capacity is often correlated with a bigger surface area.^157^ The surface area of adsorbents is essential for their adsorption abilities due to its impact on the adsorption strength with adsorbate.^158,159^ Moreover, during adsorption, changes in morphology and cracks could increase the effective surface area, thereby enhancing the adsorption capacity.^160^

The adsorption isotherms provide insights into the interactions of the adsorbed molecules with the surface of the adsorbent material. These interactions are commonly characterized using Langmuir, Freundlich, and Temkin isotherms.^161^ Physisorption is based on the assumption that it occurred in a single layer on a uniform surface with a finite number of sites to adsorb, to obtain a homogeneous surface assumption for adsorption, as described by the Langmuir model.^162^ The sorption process is nonideal and heterogeneous, and therefore better described by the Freundlich model.^163^ The Freundlich model does describe the multilayer adsorption and the non-ideal adsorption on the heterogeneous surfaces.^164^ The Temkin and Dubinin–Radushkevich models are also employed for adsorption studies. They are commonly employed to explain adsorption behavior, in which they have heterogeneous energy distributed over the nonlinear surface.^165^

Several models have been proposed to describe and predict the adsorption kinetics based on the experimental data. Common kinetic models employed to scrutinize adsorption phenomena comprise pseudo-first-order (PFO), pseudo-second-order (PSO), Avrami kinetic models, *etc.*^166^ These models help identify the rate controlling step of the adsorption process and provide an insight into the adsorption order of the individual molecules.^167^ Different quantitative models of mathematics play an important role in the analysis of adsorption kinetics.^168^ Theoretical sorption kinetics models, especially those describing surface-response processes, have been extensively reviewed. These reviews have demonstrated that the understanding of kinetics is crucially important for optimizing the design of all types of systems.^169^ The assessment and application of adsorption kinetic models is essential to understand the mechanisms and also help in trajectory prediction and improvement of adsorption processes.

**Fig. 4:**
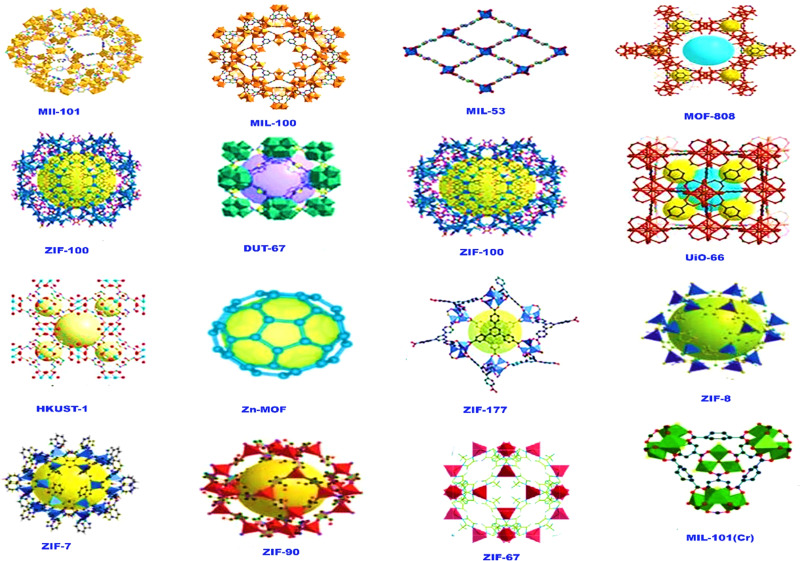
Some of the reported MOFs for arsenic removal.

Different aspects of adsorption and their impact on the treatment methods are reviewed. Crucial factors include removal efficiency, synthesis methods, specific surface area, pH, initial ion concentrations, adsorption kinetics, and isotherms. These aspects improve our understanding of the adsorption process and speed up the design of customized water treatment systems. Some of the most important MOFs reported for the removal of arsenic from water are listed in Fig. 4.

Following are the key MOF adsorbents used for arsenic removal and their key properties.

### 4.1 MOFs categorized by metal type

Since MOFs are made of metals with organic linkers, the strong interaction of the metals with the arsenic species leads to metal-based MOFs as efficient adsorbents for arsenic removal. Metal-categorized organic frameworks, such as those based on zirconium, iron, zinc, aluminum, and copper, have high adsorption capacity, stability, and reusability, making them promising materials for arsenic removal from water. Following are the key metal-based MOFs used for arsenic removal.

#### 4.1.1 Zirconium-based MOFs

Zirconium-based MOFs with outstanding stability, high surface area, and adjustable structural features have attracted considerable interest for the removal of arsenicals. These types of MOFs consist of zirconium oxide clusters, which enhance the surface area and offer multiple functional sites for the removal of arsenic species. UiO-66 MOFs exhibit enhanced retention due to the formation of Zr–O–As coordination bonds, facilitated by both hydroxyl groups as benzenedicarboxylate ligands. Moreover, the adsorption performance of Zr-MOFs can be further improved for the selective arsenic removal by functionalizing the Zr-MOFs, to achieve excellent results.^69^

The stable Zr-based MOFs demonstrated an impressive As(v) adsorption capacity of 278 mg g^−1^ within the pH range of 4–9. Mechanistic investigations confirmed that the Zr–OH sites were replaced in a spatially mediated fashion *via* ligands, as well as interactions with dissociated Zr–O linkers. After five adsorption–desorption cycles, the materials showed 90% regeneration rate, indicating strong potential for long-term arsenic removal.^170^ Another study investigated the selective adsorption of organic arsenic acids (OAAs) using seven Zr-based MOFs, where MOF-808F was identified as the most effective adsorbent, demonstrating adsorption capacities of 621.1 mg g^−1^ and 709.2 mg g^−1^ for ASA (arsanilic acid) and ROX (roxarsone), respectively. The exceptional selectivity, recyclability and resistance were attributed to the π–π stacking, hydrogen bonding, and electrostatic interactions within the MOF structure.^171^

The performance of Zr-MOFs was further enhanced by their structural characteristics. UiO-66(Zr) proved exceptional applicability in water treatment by attaining an As(v) adsorption capacity of 380 mg g^−1^ at pH 2. The arsenic (As^3+^) removal efficiency was significantly enhanced after amine (NH_2_) and thiol groups were introduced.^172^ Furthermore, among the Zr-MOFs, La/Zr-BDC-4 MOFs exhibited an excellent adsorption capacity of 694 mg g^−1^ under suitable working conditions.^36^ UiO-66-36TFA nanoparticles, synthesized by using monocarboxylic acid modulators, enhanced As(v) adsorption up to 200 mg g^−1^ at neutral pH values, the highest reported for such conditions. The free Lewis acid sites doubled the arsenate uptake compared with normal UiO-66 while maintaining high selectivity and recyclability, highlighting the potential of Zr-MOFs as regenerable adsorbents.^39^

The structural tunability of Zr-MOFs has increasingly shifted focus towards applications in electrocatalysis, extending beyond traditional adsorption mechanisms used in water treatment. The research suggested that modifying the metal nodes and organic linkers in Zr-MOFs could enhance pollutant adsorption; however, it did not provide direct evidence to validate this claim.^173^ Finally, the development of rapid synthesis techniques has resulted in the production of highly effective Zr-MOFs. MOF-808 (Zr_6_O_4_(OH)_4_(BTC)_2_(HCOO)_6_) prepared in 5 min using a household microwave oven exhibited superacidity and an arsenic adsorption capability of 24.83 mg g^−1^. After five cycles, the material maintained 82.10% removal efficiency, demonstrating its ability as a regenerable adsorbent for arsenic.^87^ Zr-MOFs exhibit great promise for arsenic removal owing to their high stability, tunable surface chemistry, and enhanced adsorption capacities, making them remarkable candidates for advanced water purification technologies.

#### 4.1.2 Iron-based MOFs

Iron-based MOFs have attracted widespread attention as excellent arsenic adsorbents for their tunable porosity, structural flexibility, and accessibility of metal sites. These characteristics facilitate robust coordination interactions with arsenic species, leading to remarkable adsorption capacity and selectivity.

MIL-100(Fe) exhibited a high porous structure with numerous accessible Fe^3+^ sites, providing abundant binding sites for arsenic species. The adsorption of both As(iii) and As(v) ions involved the formation of inner sphere complexes *via* the Fe–O–As and ligand exchange mechanism, where arsenic species replaced coordinated hydroxyl or water molecules on Fe centers. This structural flexibly and active sites contribute to the high affinity and arsenic adsorption capacity of the material.^174^ Another study supported the important role of coordination bonds between the metal centres of the MOFs and the arsenic in the solution. The efficiency of such adsorbents could be attributed to the volume of organic ligands and dispersion interactions between them.^60^ Recent studies highlighted invocative designs such as redox-active Fe-MIL-88B-Fc, which oxidized toxic As(iii) to less harmful As(v) and achieved a very impressive uptake capacity of 110 mg g^−1^*via* both the synergistic action of their adsorptive and redox process.^152^

Iron-based MOFs also demonstrated exceptional stability and reusability. MIL-53(Fe) still maintains its crystalline structure post adsorption, showing only marginal efficiency loss after regeneration.^175^ Similarly, hydrothermally synthesized DETA-Fe-BTC achieved rapid arsenic removal (90.5 mg g^−1^ in 5 s) with an adsorption capacity of 1748.50 mg g^−1^ at pH 10, attributed to the robust Fe–O–As bonding.^176^ While MIL-101(Fe) efficiently adsorbs diverse arsenic species (*e.g.* ROX, DMA) *via* Fe–O–As coordination and π–π stacking, its strong arsenic-MOF interaction may limit its reusability.^177^ These advancements underscore the potential of iron-based MOFs for scalable water treatment, particularly in the region with severe arsenic contamination.

#### 4.1.3 Zinc-based MOFs

Zinc-based MOFs such as zeolitic imidazolate framework-8 (ZIF-8) have emerged as promising materials for arsenic removal from water. ZIF-8 features a robust tetrahedral structure composed of zinc ions with 2-methylimidazole linkers forming a solidate topography. This configuration imparts high thermal stability and chemical resistance, making ZIF-8 suitable for aqueous solutions. ZIF-8 exhibited high capacity for arsenic and maintained the performance even at neutral pH levels.^48,178^ Wider affinity towards various adsorbates would further enhance the adsorption capacity of the framework, broadening the potential for environmental applications.

A comparative study of As(v) adsorption using Zn-MOF-74 synthesized *via* a room-temperature precipitation method (RT-Zn-MOF-74) and a solvothermal method (HT-Zn-MOF-74) also highlighted the important role of crystal size affecting the adsorption performance. The nanosized RT-Zn-MOF-74 combined the advantages of smaller particle size and better dispersion, leading to a higher adsorption capacity of 99 mg g^−1^ than the 48.7 mg g^−1^ adsorption capacity of HT-Zn-MOF-74 for As(v). The findings show that tuning the crystal size enhanced the adsorption capacities of the MOFs.^38^

ZIF-8 with its high porosity and a large surface area is well suited for effective arsenic adsorption. Because of its specific structural properties, ZIF-8 can coordinate with arsenic species in both oxidation states, *i.e.* As(v) and As(iii).^49,179^ ZIF-8@Fe_3_O_4_, a composite material consisting of Fe_3_O_4_ nanoparticles encapsulated by ZIF-8 with a surface area of 316 m^2^ g^−1^, achieved an adsorption capacity of 116.114 mg g^−1^ under acidic condition (pH 3).^77^ Hierarchically porous ZIF-8 (HP-ZIF-8), synthesized using ZnO nanoparticles as both Zn source and pore template, exhibited a significantly enhanced As(iii) adsorption capacity of 104.9 mg g^−1^, almost doubled compared to that of the conventional ZIF-8.^49^ Similarly, a novel Zn-MOF was prepared using Zn^2+^ and 3-amino-5-mercapto-1.2,4-trizole for the efficient removal of heavy metals, including arsenic. At optimal pH levels, the highest adsorption capacity for Pb, Hg, and As was 1097 mg g^−1^, 32 mg g^−1^ and 718 mg g^−1^, respectively. The characterization results demonstrated that the stability, surface area, and adsorption results followed Langmuir and pseudo-second-order models.^133^

The Zn-MOF-74 crystal demonstrates remarkable adsorption ability for As(v) (325 mg g^−1^) and As(iii) (211 mg g^−1^). These values represent a record-high As(v) removal and the second-highest removal efficiency for As(iii) at publication time. The coordination interactions of Zn(ii) ions with the arsenic species primarily drive the elimination process.^180^ The stability study of ZIF-8 in aqueous medium, which retain performance even after several regeneration cycles, highlighted its suitability for practical applications.^179^

#### 4.1.4 Other metal-based MOFs

Copper, aluminum, cerium, and lanthanum containing MOFs have also been reported to be significant in arsenic removal, alongside extensively researched examples of zirconium, iron, and zinc-based MOFs. The use of different adsorption processes including electrostatic interactions, coordination bonding, and ligand exchange in these MOFs further improves their capability for water filtration.

Copper-based MOFs have shown potential to be effective adsorbents, because of their tunable porosity, high surface area, and presence of accessible open metal sites that significantly enhanced the adsorption capacity. Their excellent structural stability, combined with strong coordination interactions with arsenic ions, leads to increased adsorption capacities, making them highly effective in wastewater treatment applications.^181^ In addition, the geometric structure of copper-based MOFs varies with the selection of ligands, which can effectively improve its adsorption performance.^182^ Their cost-effectiveness and natural abundance make them attractive candidates for scalable water treatment options.^183^

Aluminum-based MOFs are reported to be attractive, due of their structural tunability and high surface area, thus facilitating effective adsorption of arsenic. Aluminum has two coordination sites, allowing the formation of strong interactions with arsenate and arsenite, which enhanced their affinity and binding strength. For instance, MIL-53(Al) showed a high maximum adsorption capacity of 105.6 mg g^−1^ under optimized conditions while retaining efficiency even at low arsenic concentrations. The selective arsenic capturing by these materials may be attributed to main adsorption mechanisms such as hydrogen bonding or electrostatic interactions, with minimal competition by other anions.^184^

The cerium and lanthanum-based MOFs have recently shown great potential for As(iii) or As(v) removal, owing to their higher adsorption capacities and selective binding mechanisms. For example, UiO-66(Ce) showed an exceptional adsorption capacity of 308 mg g^−1^ for As(v), where chemisorption was the predominant mechanism, possessing great stability and reusability.^185^ Furthermore, Ce-MOF-66 and Ce-MOF-808 showed impressive As(v) and As(iii) adsorption capacities of 355.67 mg g^−1^ and 402.10 mg g^−1^, respectively, with ligand exchange and unsaturated metal sites being involved in the adsorption process.^186^ A list of different MOFs categorized by metal type and their characteristics for arsenic removal are presented in Table 2.

**Table 2:** Different metallic MOFs for arsenic removal and their dimensions

Adsorbent	Synthesis method	Surface area m^3^ g^−1^	Target ion	Initial ion conc.	pH	Adsorption capacity mg g^−1^	Adsorption model		Ref. #
							Isotherm	Kinetic	
MOF-808	Microwave irradiation	NA	As^5+^	5 mg L^−1^	4	24.83	NA	PSO	87
MOF-808F	Modulated solvothermal	811–2393	ASA	5 mg L^−1^	4	621.1	Langmuir	PSO	171
			ROX			709.2			
MOF-808F			ASA			649.4			
			ROX			641			
La/Zr-BDC-1	One pot hydrothermal	252.40	As^5+^	755 mg L^−1^	7	542.8	Langmuir	PSO	36
La/Zr-BDC-4		42.44				694			
Fe-MOF	Hydrothermal	128.3	As^5+^	100 mg L^−1^	7	70.02	Langmuir	PSO	187
Zr-MOF		290.4				85.72			
La-MOF		61.8				114.28			
Fe-MOF	Hydrothermal	128.3	As^5+^	100 mg L^−1^	7	70.02	Langmuir	PSO	187
Zr-MOF	Hydrothermal	290.4	As^5+^	100 mg L^−1^	7	85.72	Langmuir	PSO	187
La-MOF	Hydrothermal	61.8	As^5+^	100 mg L^−1^	7	114.28	Langmuir	PSO	187
Fe-BTC MOF	Batch experiment	NA	As^5+^	10 mg L^−1^	7.5	0.975	NA	NA	188
MIL-100(Fe)	Microwave assisted hydrothermal	720	As^3+^	2.5 mg L^−1^	8.57	35.2	NA	NA	174
			As^5+^			19.2			
Ni-MOFs	Hydrothermal	41.51	As^5+^	20 mg L^−1^	3	133.9	Langmuir	PSO	189
NiO/Ni@C400		74.78	As^5+^			454.9	Langmuir	PSO	
Zr-MOF (SUM-8)	Solvothermal	3268	As^5+^	100 mg L^−1^	2	152.2	Langmuir	PSO	190
Fe-MIL-88B-Fc	Hydrothermal	186.4	As^3+^	NA	7.7	110	NA	NA	152
Nano-{Fe-BTC}	Direct method	427.2	As^3+^	10 mg L^−1^	9	12.20	Langmuir	PSO	191
			As^5+^	10 mg L^−1^		13.61			
Basolite^®^300		840	As^3+^	10 mg L^−1^	10	11.72			
			As^5+^	10 mg L^−1^		16.16			
MOF-76(Y)-Ac	Solvothermal	980.33	As^5+^	50 mg L^−1^	9–11	201.46	Langmuir	PSO	192
MOF-76(Y)		919.49				187.78	Langmuir	PSO, PFO	
HP-ZIF-8	*In situ* vapour deposition	234.37	As^3+^	100 mg L^−1^	9	105	Langmuir	PSO	49
ZIF-8		936.3				58.55			
Zn-MOF	Solvothermal	89.8	As^5+^	250 mg L^−1^	5	32	Langmuir	PSO	133
Zn-MOF-74	Solvothermal	604	As^3+^	450 mg L^−1^	12	205	Langmuir	PSO	180
			As^5+^	300 mg L^−1^	7	325			
MIL-53(Al)	Hydrothermal	920	As^5+^	8 mg L^−1^	8	105.6	Langmuir	PSO	184
HT-Zn-MOF-74	Precipitation, solvothermal	1201	As^5+^	150 mg L^−1^	7	48.7	Langmuir	PSO	38
RT-Zn-MOF-74		690	As^5+^			99			
AUBM-1 MIL-53(Al)	Sonochemical, solvothermal	310	As^5+^	200 mg L^−1^	7.6	103.1	Langmuir	PSO	50
Fe based MIL-88A(microrods)	Hydrothermal	NA	As^5+^	100 mg L^−1^	5	145	Langmuir	PSO	193
MOF-808	Solvothermal	NA	As^3+^	300 mg L^−1^	5	161.1	Langmuir	PFO	194
La@MOF-808	Solvothermal		As^3+^		5	325.7	Langmuir	PSO	
MIL-88A-Fe	Direct mixing method	6.90	As^3+^	25 mg L^−1^	9.5	24.86	Langmuir	6.90	195
ZIF67	NA	NA	As^5+^	10 mg L^−1^	6	62.98	Langmuir	PSO	196
UiO-66(Ce)	Ambient temperature	858	As^5+^	300 mg L^−1^	6	70	Langmuir	PSO	185
Fe-UiO-66-M	Thermal solvent	980	As^5+^	400 mg L^−1^	3	337	Langmuir	PSO	197
Ce-MOF-66	Ligand tuned solvothermal	965.29	As^3+^	100 mg L^−1^	10	5.52	Langmuir	PSO	186
			As^5+^		2.5	355.67			
Ce-MOF-808		335.12	As^3+^		10	402.10	Langmuir	PSO	
			As^5+^		2.5	217.80			
MIL-88A(Fe)	Room tem. solvothermal	13.39	As^3+^	150 mg L^−1^	11	164	Langmuir	PSO	198
			As^5+^		11	126.5			
			ASA		11	427.5			
			ROX		5	261.4			
MOF (L_2_O_3_Co_2_)	Solvothermal & calcination	7.5	As^5+^	175 mg L^−1^	7	221.24	Langmuir	PSO	199
MIL-88B(Fe)	Optimize scalable	41.68	As^5+^	20 mg L^−1^	7	129	Freundlich	PSO	200
MIL-100(Fe) pristine	Solvothermal	844	As^5+^	10 mg L^−1^	8	70	Langmuir	PSO	201
MIL-100(Fe)-BA1 defective		1081				174			
Activated MIL-88A	Solvothermal	210	As^5+^	100 mg L^−1^	7	347	Langmuir	PSO	202
			_P_-ASA		6	281			
			ROX		4	252			
			DMA		7	96.8			

#### 4.1.5 Bimetallic MOFs

Bimetallic MOFs are emerging candidates for the removal of arsenic. These crystalline, solid-state materials possess highly reticulated frameworks, offering high surface areas and tunable pore sizes. These characteristics enhance the accessibility of water and arsenic ions to the internal network, significantly improving the removal of water contaminants including arsenic species.^120^ The incorporation of multiple metal ions into the MOF structure enhances the adsorption efficiency in comparison to the single metal frameworks.^203^

Fe/Mn MOFs, synthesized *via* a hydrothermal method, demonstrated high adsorption capacities of 344.14 mg g^−1^ for As(iii) and 228.79 mg g^−1^ for As(v), respectively, within just 30 minutes. These capacities were much higher than those of MIL-88A, exhibiting strong coordination interactions that facilitated arsenic binding. The presence of Fe and Mn played a key role in catalyzing the reoxidation of As(iii) to As(v) and enhanced the overall removal efficiency.^204^ Fe/Mg-MIL-88B MOFs are efficient in removing arsenic, with tunable Fe/Mg ratios exhibiting enhanced As(v) sorption with a remarkable uptake capacity of 303.6 mg g^−1^, fast sorption kinetics, high capacity and excellent stability in multiple sorption/desorption cycles, making them suitable for arsenic decontamination in water.^63^ Another Fe/Mn-based MOF, Fe_0.3_Mn_0.3_-MOFs, designed for synergistic arsenic removal *via* adsorption and PMS(peroxymonosulfate)-coupled oxidation, achieved 98% removal of As(iii) in natural contaminated water. This system displayed impressive recyclability, retaining 78% efficiency even after five cycles.^205^

La_0.75_Fe_1.0_-MOF-based heterometallic MOFs, incorporating lanthanum-doped iron, were prepared for enhanced arsenic removal, which exhibited high adsorption capacities of 242.28 mg g^−1^ for As(v) and 307.15 mg g^−1^ for phosphate, following an impulsive monolayer adsorption mechanism, driven by electrostatic connections and complexation.^206^ Furthermore, a Fe–Ti heteroatom-based MOF, MIL-125(Ti, Fe), exhibited a remarkable potential for As(v) removal, achieving 99.3% removal efficiency from 10 ppm water, and reduced As(v) to just 3 ppb in breakthrough tests. The adsorption process involved the formation of Fe–O–As complexes and oxygen vacancies, which confirmed its effectiveness for the removal of arsenic.^207^

Fe/Co bimetallic MOFs integrated with peroxymonosulfate (PMS) proved to be effective for the oxidation of As(iii) and adsorption of As(v) in DOM (dissolved organic matter)-rich, high-arsenic groundwater. The Fe/Co MOF-PMS system efficiently addressed interference from DOM through non-radical-driven oxidation and chemisorption, resulting in efficient arsenic removal despite the presence of humic acid.^208^ An asymmetric bimetallic MOF, UiO-66(Fe/Zr), exhibited outstanding performance in the removal of arsenic from water with high adsorption capacities for As(v) (204.1 mg g^−1^) and As(iii) (101.7 mg g^−1^), with a fast kinetics (equilibrium in 30 min). Strong chemisorption with Fe/Zr–O–As linkages yielded minimal arsenic leaching (99%) from real water samples, indicating its potential for advanced water purification.^209^

Bimetallic metal–organic frameworks, particularly those including Fe/Mn, Fe/Ti, Fe/Co, and Fe/Zr, have shown significant advancements in the extraction of arsenic from aqueous solutions. Their substantial adsorption capabilities, rapid kinetics, recyclability, and ability to function in complex aquatic settings make them very attractive materials for large arsenic cleanup initiatives. Important bimetallic MOFs and their properties used for arsenic removal are shown in Table 3.

**Table 3:** Different bimetallic MOFs for arsenic removal and their dimensions

Adsorbent	Synthesis method	Surface area m^3^ g^−1^	Target ion	Initial ion conc.	pH	Adsorption capacity mg g^−1^	Adsorption model		Ref. #
							Isotherm	Kinetic	
Fe/Mn-MOF	Hydrothermal	38.91	As^3+^	50 mg L^−1^	11	314.14	Langmuir	PSO	204
			As^5+^		3–11	228.79	Langmuir	PSO	
FeMn-MOF-74	One pot solvothermal	45.82	As^3+^	180 mg L^−1^	7	161.6	Langmuir	PSO	210
Fe/Mg-MIL-88B (0.5)	One step solvothermal	360	As^5+^	200 mg L^−1^	7	303.6	Langmuir	PSO	63
La_0.75_Fe_1.0_-MOF	Solvothermal	53.7	As^5+^	100 mg L^−1^	7	307.15	Langmuir	PSO	206
MIL-125(Ti, Fe)	Solvothermal		As^5+^	10 mg L^−1^		99.9%	Langmuir	PSO	207
Fe_0.3_Mn_0.3_-MOFs	Hydrothermal	71.75	As^3+^	20 mg L^−1^	11	98%	Langmuir	PSO	205
UiO-66(Fe/Zr)	One step hydrothermal	498.33	As^3+^	200 mg L^−1^	7	101.7	Langmuir	PSO	209
			As^5+^			204.1			
ZrFc-MOF/PMC	Solvothermal	304.66	As^3+^	120 mg L^−1^	7	111.34	Langmuir	PSO	211
ZrFc-MOF						59.59			
Fe_2_Co_1_MOF-74	Solvothermal	147.82	As^3+^	100 mg L^−1^	4.3	266.52	Langmuir	PSO	212
			As^5+^			292.29			
MOF-ZIF67/ZIF8	Coprecipitation-solvothermal	950	As^5+^	50 mg L^−1^	6.5	71.4	Langmuir	PSO	213
Zr-MOFs	Solvothermal	NA	As^5+^	100 mg L^−1^	350	278	Langmuir, Freundlich	PFO	170
C-Fe/Ni NPs	Green synthesis	345.63	As^5+^	1.5 mg L^−1^	6	1.17	Langmuir	PSO	214
Co_*x*_Fe_3−*x*_O_4_	Solvothermal, calcination	NA	As^3+^	100 mg L^−1^	7	119	Langmuir	PSO	215

### 4.2 Hybrid MOFs

Hybrid Metal–Organic Frameworks (MOFs) are advanced material compounds made from organic ligands and inorganic metal ions or clusters that are coordinated *via* coordination bonds and are ideally porous crystalline materials. The utilization of hybrid MOFs in water solutions as a remediation of heavy metals has emerged as an important research direction due to their fantastic structural properties and high adsorption capacity. With the addition of other functional materials such as magnetic nanomaterials, polymers, graphene oxide (GO) and metal oxides, these adsorbents increased their efficiency and stability towards arsenic removal.^22,51^

In recent years, tremendous progress has been made in hybrid materials combining MNPs embedded within magnetic hybrid MOFs, which contributed to the ease of separation and recyclability, rendering them very attractive for the treatment of water. Fe_3_O_4_@ZIF-8 is a prominent example of MOFs, which has demonstrated high efficiency in trapping arsenic ions from water while providing easy recovery due to its magnetic property.^77^ Likewise, Fe_3_O_4_@UiO-66 prepared through a two-step solvothermal procedure exhibited a substantial adsorption capacity of 73.2 mg g^−1^ for arsenate, adsorption followed the pseudo-second order kinetics, and fitted well to Freundlich isotherm models. Due to its hard surface area, thermal, and notable magnetic properties, it has shown importance in wastewater remediations.^216^ CoFe_2_O_4_@MIL-100(Fe), another excellent hybrid material, exhibited adsorption capacities for As(v) and As(iii) of 114.8 mg g^−1^ and 143.6 mg g^−1^, respectively. Its high removal efficiency of arsenic from natural water is attributed to Fe–O–As and hydrogen bond interactions.^217^

The incorporation of polymers into MOFs has increased the mechanical strength and operational stability to a level suitable for practical applications in the real world. Porous hybrid adsorbent beads were prepared using chitosan solvogel matrix combined with MIL-100(Fe), achieved a remarkable selectivity (99%) and an efficiency of 99% for both As^5+^ and As^3+^.^218^ This eco-friendly approach makes them appealing for drinking water treatment. Similarly, Zn-MOF/PVA nanofibrous composites, with high arsenic adsorption capacity, maintained their efficiency even after several adsorption–desorption recycling cycles, ensuring its sustainability.^51^

Graphene oxide (GO) has been incorporated into MOFs to improve their dispersibility, water stability, and adsorption performance. The great surface area of MOFs coupled with the outstanding adsorption properties of GO, made MOF/GO composites a widely recognized adsorbent for the removal of heavy metals.^219^ FeZr-MOFs/GO nanocomposite, developed by doping MIL-101(Fe) with Zr(iv), showed an excellent adsorption capacity of 91.69 mg g^−1^ for As(v). The presence of Zr improved water stability and adsorption capability through both chemical complexation and hydrogen bonding, resulting in substantial arsenic removal across a wide pH range.^220^ A Prussian Blue Analogue (PBA)@GO membrane, synthesized for arsenate removal through Fenton-like reactions, showed good stability for over 80 h and also effectively removed both arsenic and total organic carbon.^221^ Furthermore, the MWCNTs/MIL-53(Fe) nanocomposite designed for water stability exhibited effective removal of As(v) from groundwater. Under optimum conditions, this nanocomposite adsorbed 27.24 mg g^−1^ of As(v) within just 20 min, in the pH range of 3–10. The adsorption mechanism was dominantly controlled by hydrogen bonding, electrostatic attraction and chemical complexation, which together contributed for enhanced As removal.^222^

Hybrid MOFs containing metal sulfides and metal oxides have been developed to improve arsenic adsorption. FeS_*x*_@MOF-808, designed for As(iii) removal from wastewater, exhibited an outstanding adsorption capacity of 203.28 mg g^−1^ at pH 7, by integrating the high porosity of MOF-808 with abundant FeS_*x*_ active sites. The adsorption mechanism involved a synergetic process of adsorption, co-precipitation, and Fe–S bond cleavage, ensuring stable arsenic sequestration over a wide pH range.^223^ Another high-performance material, δ-MnO_2_@Fe/Mg-MIL-88B, demonstrated superior As(iii) removal over a pH range (2–10) by combining the oxidizing power of δ-MnO_2_ with the high As(v) uptake capacity of Fe/Mg-MIL-88B.^71^ Similarly, the δ-MnO_2_@Fe/Co-MOF-74 composite achieved an impressive adsorption capacity up to 300.5 mg g^−1^ for As(iii), demonstrating high stability even in the presence of common competing ions, and the primary removal mechanism involved electrostatic adsorption and complexation, making it a strong candidate for practical arsenic remediation.^224^

Hierarchically porous MOFs exhibited ultrafast adsorption kinetics and enhanced arsenic removal efficiency. Fe-MOG/BC, an iron-based porous MOF, demonstrated an extraordinary As(v) uptake capacity of 495 mg g^−1^, making it one of the most effective arsenic adsorbents. This hybridization ensured not only more efficient adsorption but also more potential applications for the immediate continuous separation of MOFs under different environmental conditions.^59^ Another promising material, ZIF-L, a two-dimensional leaf-like zeolitic imidazolate framework, was synthesized under room-temperature conditions, which showed high adsorption efficiency for As(iii). At pH 10, it exhibited an uptake of 43.43 mg g^−1^, primarily through electrostatic interactions and inner sphere complex formation.^225^ Additionally, ZIFs with cubic, leaf-shaped, and dodecahedral shapes prepared using green methods achieved adsorption capacities of 122.6 mg g^−1^, 108.1 mg g^−1^ and 117.5 mg g^−1^ for As(iii), at pH 8.5. The substitution of zinc hydroxyls was a key factor in the surface complexation of As(iii) removal, which was certified by FTIR and XPS analysis results.^226^ Different hybrid MOFs reported for arsenic removal are illustrated in Table 4.

**Table 4:** Adsorption measurement of different hybrid MOFs for arsenic removal

Adsorbent	Synthesis method	Surface area	Target ion	Initial ion conc.	pH	Adsorption capacity mg g^−1^	Adsorption model		Ref. #
							Isotherm	Kinetic	
F-300	Hydrothermal	NA	As^5+^	45 mg L^−1^	7	169.2	Langmuir	PSO	120
Fe_3_O_4_@ZIF-8	Coprecipitation	316	As^5+^	50 mg L^−1^	3	116.11	Langmuir	PSO	77
IL-100-Fe (ChitFe5)	Sol gel and solvothermal	NA	As^3+^	80 mg L^−1^	9	28.09	Langmuir	PSO	218
			As^5+^		7	23.17			
MIL-100-Fe (ChitFe7)			As^3+^		9	35.25			
			As^5+^		7	64.45			
MOF-808	Coprecipitation & precipitation	2161	As^3+^	50 mg L^−1^	7	27.85	Langmuir	PSO	223
Fe@MOF-808		45.61			11	34.26			
FeS_*x*_@MOF-808		253.54			9	73.60			
Zn-MOF/PVA	Electrospinning	3404	As	0.016 mg L^−1^	NA	98%	NA	NA	51
Cubic ZIFs	Solvothermal	958.4	As^3+^	70 mg L^−1^	8.5	122.8	Langmuir	PSO	226
Leaf shaped ZIFs		12.7				108.1			
Dodecahedral shaped ZIFs		1151.2				117.5			
Fe-MOG/BC	Sol gel, hydrothermal	NA	As^5+^	100 mg L^−1^	6	495	Langmuir	PSO	59
Fe-MOF/BC						220.65			
CoFe_2_O_4_@MIL-100(Fe)	One pot hydrothermal	292	As^3+^	100 mg L^−1^	4–10	143.6	Langmuir	PSO	217
			As^5+^			114.8			
Fe_3_O_4_@UiO-66	Solvothermal	124.8	As^5+^	150 mg L^−1^	7	73.2	Freundlich	PSO	216
FeZr-MOFs-_0.2_/GO	Modified hydrothermal	293	As^5+^	25 mg L^−1^	6	158.6	Langmuir	PSO	220
δ-MnO_2_@Fe/Mg-MIL-88B	Hydrothermal, ultrasonication	118	As^3+^	250 mg L^−1^	6	221	Freundlich	PSO	71
MWCNTs/MIL-53(Fe)				10 mg L^−1^		24.24			222
β-MnO_2_@ZIF-8	Solvothermal	883	As^3+^	40 mg L^−1^	7	140.27	Langmuir	PSO	227
Magnetic-ZIF8	Coprecipitation	696.5	As^3+^	10.6 mg L^−1^	7	30.87	Langmuir	PSO	228
			As^5+^			17.51	Langmuir	PSO	
F-ZIF8@EMM	Precipitation, hydrothermal, sonochemical	1483.13	As^5+^	0.1 mg L^−1^	7	0.444	Thomas	NA	229
UiO-66/PAN	Modified solvothermal	NA	As^3+^	100 mg L^−1^	7	32.90	Langmuir	PSO	230
			As^5+^		7	42.17	Langmuir	PSO	
Fe_3_O_4_@ZIF-8	Solvothermal & *in situ* growth	1133	As^3+^	27 mg L^−1^	8	100	Langmuir	PSO	231
Zn-BDC@CT/CNT	Modified hummers	310.2	As	50 mg L^−1^	4	80	Freundlich	Elovich	232
Zn-BDC@CT/GO		327.9				128.20			
MIL-101 (Fe) @CM	*In situ* hydrothermal	90	As^5+^	40 mg L^−1^	7	70.4	Langmuir	PSO	233
MIL-101(Fe)		2200				322.6	Langmuir	PFO	
MIL101(Fe)@PET	Microwave method	0.131	As^5+^	100 mg L^−1^	5	240	Langmuir	PFO	234
			ROX	200 mg L^−1^	6	476.5			
			ASA		3	320.6			
ZFM@UiO-66	Sol gel electrospun	171	As^3+^	400 mg L^−1^	2–8	144	Langmuir	PSO	235
MIL-88B/MnO_2_/GAC	*In situ* reduction	NA	As^3+^	20 mg L^−1^	7	15.13	Langmuir	PSO	236
γ-Fe_2_O_3_@CFT-1	Iono-thermal	1049	As^3+^	10 mg L^−1^	7	198	Langmuir	PSO	237
			As^5+^			102			
AC@Fe-MOF	Hydrothermal	158.29	As^5+^	300 mg L^−1^	9	1069	Freundlich	PSO	238
HFeO@MIL-100	Solvothermal	721	As^5+^	75 mg L^−1^	8–10	140.6	Freundlich	PSO	239

### 4.3 Functionalized MOFs

Functionalization is an integral way to give tightly function groups that may allow the binding of arsenic ions. Recent studies have focused on different functionalized MOFs with excellent arsenic uptake capacities, making them remarkable candidates for water treatment processes. These various functional groups serve to facilitate not only the binding efficiency but also the tethering of additional species.

Amine functionalization has been widely studied due to its ability to improve arsenic adsorption. For example, diethylenetriamine-functionalized MIL-53(Fe) (MIL-DETA-*n*) achieved a maximum As(v) adsorption capacity of 137.5 mg g^−1^, while minimizing Fe leaching below 0.3 mg L^−1^. The adsorption kinetics followed the pseudo-second-order model and the isotherm followed the Langmuir isotherm, signifying efficacy in eradicating arsenic from surface and groundwater.^240^ Similarly, NH_2_-MIL-101(Fe) exhibited high removal efficiency for As(v) and As(iii) in a wide pH range with adsorption capacities significantly surpassing those of unmodified MIL-101(Fe). This enhanced performance is due to the high Fe content, high Fe^3+^/Fe^2+^ ratio, and increased surface area.^241^ Functionalization and doping further enhanced the arsenic removal performance. Gadolinium-doped MIL-10-NH_2_ (0.75GF-MILN) achieved high arsenic(v) and phosphorus adsorption capacities of 220.7 mg g^−1^, and 112.8 mg g^−1^, respectively, across a wide pH range, with electro-assisted regeneration preserving structural integrity.^73^

Chelating agents enhance the specificity and strength of arsenic adsorption from water. In dihydrotetrazine (DHTZ)-decorated UiO-66 (Zr) MOFs, the chelation process greatly improved As(v) removal efficiency by forming strong coordination bonds with arsenic species.^57^ Additionally, cerium-doped MIL-101-NH_2_ (1Ce-MIL-101-NH_2_) was prepared for the simultaneous removal of phosphate and As(v), achieving adsorption capacities of 341.5 mg g^−1^ for phosphate and 249 mg g^−1^ for As(v). The materials effectiveness was credited to electrostatic attraction and complexation between Fe/Ce and oxyanions, demonstrating exceptionally well selectively even in multi-anion solutions.^54^

Iron-based MOFs have received considerable attention for their tunable properties and synergistic effects, and functionalization allows improving its functions. Spindle-like morphology was obtained by the one-step strategy of Fe/Mg-MIL-88B MOFs and enhanced As(v) removal (303.6 mg g^−1^) by dual-metal interactions. The hybrid variant, as compared to the monometallic Fe-MIL-88B, showed better adsorption capacities and rapid sorption kinetics, demonstrating great potential for water treatment.^63^ The ZnAl-LDHs/NH_2_-MIL-125 composite, synthesized from the *in situ* growth of Ti-based MOFs in Layered double hydroxides (LDH), showed an outstanding As(v) adsorption capacity of 1634.0 mg g^−1^ at pH 10. The porous structure of MOFs and high adsorption affinity of LDHS were leveraged, as demonstrated by the Langmuir isotherm and pseudo-second-order kinetics.^242^

Polymer composites represent an attractive approach towards enhancing heavy metal binding in MOFs. MOF@polymer composites were constructed using a foam-like solid structure with a large abundance of high-density distribution of different Zr, Zn, Co, Al, and Cr heavy metals embedded in the polymer matrix. Hydrophobic polymers promoted adsorption behaviour by providing a more adequate interfacial contact between the MOF surfaces and arsenic species in the aqueous solution.^243^

Nanocomposites incorporating MOFs with other materials have demonstrated exceptional arsenic removal efficiencies. MIL-100(Fe)/reduced graphene oxide (rGO)/δ-MnO_2_ nanocomposites achieved high adsorption capacities of 192.67 mg g^−1^ for As(iii) and 162.07 mg g^−1^ for As(v) at pH 2. The material remained stable across a wide pH range and showed excellent reusability, with adsorption mechanisms dominated by electrostatic interactions, redox reactions and surface complexations.^244^ Additionally, magnetic Fe_3_O_4_/GO nanocomposites were prepared to reduce arsenic contamination of groundwater. The improved nanocomposite exhibited excellent As(v) removal efficiency, accessible magnetic separation, and high pH stability. The enhanced adsorption is due to the synergistic effect of chemical complexation, hydrogen bonding, and electrostatic interactions.^245^

Another novel MOF, 2HAP=N-MIL-88 (Fe), exhibited a high As(iii) adsorption capacity of 265.5 mg g^−1^ with excellent recyclability. The material retained strong adsorption performance over multiple cycles, with optimal adsorption occurring at pH 4 *via* chemisorption mechanism.^246^ Furthermore, Ce-5-SIP-MOF, synthesized by using sodium isophthalate-5-sulfonic acid as an immobilized ligand, demonstrated a high As(v) adsorption capacity of 170.28 mg g^−1^. The composite efficiently reduced arsenic content in wastewater to meet environmental standards through a combination of Ce^3+^ and HAsO_4_^2−^ coordination, electrostatic attraction, and hydrogen bonding.^247^

Sustainable approaches have also been explored, including MOFs synthesized from waste materials. Fe-MOF, Zr-MOF, and La-MOF derived from waste PET bottles exhibited arsenic adsorption capacities of 70.02 mg g^−1^, 85.72 mg g^−1^, and 114.28 mg g^−1^, respectively. The materials showed excellent regeneration potential and low toxicity, thus proposing them as potential and eco-friendly adsorbents for the removal of arsenate from water.^187^ Beyond adsorption applications, functionalized MOFs hold great potential for arsenic detection. Chelation mechanism enables the selective detection of As(iii), the most toxic and prevalent arsenic species in contaminated water. Surface functionalization strategies enhance the MOF selectivity, allowing the fine-tuning of adsorption properties for targeted ion capture.^248^ This approach not only retains the skeletal structure of coordination oligomers but also imparts hydrophobicity to facilitate the selective enrichment of arsenic species.

Functionalized MOFs' variable chemical characteristics, high adsorption capabilities, and pH stability make them promising arsenic removers. Increasing amine-functionalization, chelation, bimetallic structures, polymer composites, and nanocomposites has improved MOFs for arsenic cleanup. Table 5 presents the list of latest MOFs reported for arsenic removal.

**Table 5:** Functionalized MOFs for arsenic removal and their properties

Adsorbent	Functional groups	Synthesis method	Surface area	Target ion	Initial ion conc.	pH	Adsorption capacity	Adsorption model		Ref.
								Isotherm	Kinetic	
Amino-MIL-68(Al)	–NH_2_, –OH, –COOH	Solvothermal	1170.9	As^5+^	50 mg L^−1^	3	74.29	Langmuir	PSO	53
Gly@UiO-66(Zr)	–NH_2_, –SH	Solvothermal, post-synthetic modification	581.4	As^3+^	200 mg L^−1^	8	301.4	Langmuir–Freundlich	PSO	46
Cys@UiO-66(Zr)			459			8	206.2	Langmuir		
Mer@UiO-66(Zr)			482.4			8	239.8	Langmuir		
Fe-MIL-88B@PGC20%	–COOH, –COO^−^, Fe–O	Hydrothermal & calcination	134.57	As^3+^	252 mg L^−1^	3	321	Langmuir	PSO	249
				As^5+^	284 mg L^−1^	9	212	Langmuir	PSO	
S-CuLao@UIO-66	–COOH, –OH, –SH *etc.*	Post synthetic modification	NA	As^3+^	30 mg L^−1^	6	171	Langmuir–Freundlich	PSO	250
Fe_3_O_4_@MIL-101(Cr)	–OH, –COO, Fe^3+^	Hydrothermal	3200	As^5+^	10 mg L^−1^	7	121.5	Langmuir	Elovich	48
Fe_3_O_4_@MIL-101(Cr)		Hydrothermal	2270	As^5+^	10 mg L^−1^	9–10	110.8	Langmuir	Elovich	48
				As^5+^	10 mg L^−1^		33.5	Langmuir	Elovich	
UiO-66(Zr) -DHTZ	–C=N–, –NH–, –COO^−^	Post synthesis *via* linker exchange	783	As^5+^	200 mg L^−1^	7	583	Langmuir	PSO	172
UiO-66 (Zr)			1140				320			
2HAP=N-MIL-88(Fe)	–NH_2_, –OH, –COO^−^	Solvothermal & post synthetic modification	180	As^3+^	210 m L^−1^	4	265.5	Langmuir	PSO	246
MOF-808-EDTA@PES	–NH_2_, –OH, –COO^−^, EDTA	Solvothermal& facile dropping	8.5	As^3+^	100 mg L^−1^	5	125	Langmuir	PSO	251
NH_2_-MIL-101@PES			13.3			6	110			
UiO 66-00	–COO^−^, Zr–OH, As–O	Solvothermal	1041	As^5+^	100 mg L^−1^	7	89.3	Langmuir	PSO	39
UiO 66-12-AA			1172				93.3			
UiO 66-36-AA			1295				103.4			
UiO 66-100-AA			1500				129			
UiO 66-12-TFA			1546				138.4			
UiO 66-36-TFA			1690				200			
DETA-Fe-BTC	–NH_2_, –OH, –COO^−^	Solvothermal	637.19	As^5+^	1000 mg L^−1^	10	1748.50	Langmuir	PSO	176
ED-ZIF-8	EDA	Precipitation	850	As^3+^	10 mg L^−1^	7	83.7	Langmuir	PSO	252
TFPOTDB-SO_3_H (sulfonated)	–SO_3_H, –C=N–	Solvothermal	190.73	As^3+^	100 mg L^−1^	8	344.8	Langmuir	PSO	253
UiO-66(Ce)-RTS	–NH_2_, –OH, –COO^−^	Ambient temperature	858	As^5+^	300 mg L^−1^	6	308	Langmuir	PSO	185
UiO-66(Ce)-NH_2_-RTS			678				70			
UiO-66-NH_2_	–NH_2_, –COO^−^	Facile solvothermal	113.4	As^3+^	100–200 mg L^−1^	9	200	Langmuir	PSO	254
				As^5+^		1, 11	71.13			
UiO-66			485.9	As^3+^		9	205			
				As^5+^		1, 11	68.21			
NH_2_-MIL-101(Fe)	–NH_2_, –OH, –COO^−^, Ce–O	One pot solvothermal	1725.7	As^3+^	300 mg L^−1^	7	153	Freundlich	PSO	241
				As^5+^			148			
H_2_N-MIL-88(Fe)	–C=N–, –OH	Solvothermal	NA	As^3+^	1 g L^−1^	8	200.94	Langmuir	PSO	255
Fe-DFC MOF	–Fc, –COO^−^	Modified hydrothermal	272.4	As^3+^	150 μg L^−1^	NA	96%	NA	NA	256
CHIPEC@UiO-66-NH_2_	–NH_2_, –OH, –COO^−^	Solvothermal	NA	As^3+^	40 mg L^−1^	8	168	Langmuir	PSO	257
				As^5+^			335			
UiO-66-NH_2_				As^3+^			254			
				As^5+^			268			
UiO-NH_2_@CC	–NH_2_, –OH, –COO^−^, C–H/C=C	Hot-press synthesis	386.94	As^5+^	100 μg L^−1^	2	3.33	Freundlich	PSO	258
Zr-MOFs (aspartic acid)	–NH_2_, –OH, –COO^−^	Hydrothermal	334.40	As^5+^	100 mg L^−1^	5–9	109.75	Langmuir	PSO	259
0.75GF-MILN	–NH_2_, –OH, –COO^−^, C–N	Solvothermal	682.5	As^5+^	100 mg L^−1^	3	220.7	Langmuir	PSO	73
MIL-101-NH_2_(Fe)			8.1				145.71			
ZnAl-LDHs/NH_2_-MIL-@125	–NH_2_, –OH, –COO^−^, M–O	Hydrothermal	115.9	As^5+^	1500 mg L^−1^	10	1634	Langmuir	PSO	242
NH_2_-UiO-66(Zr)	–NH_2_, –OH, –COO^−^, –SH	Ultrasonic, hydrothermal	859	As^3+^	50 mg L^−1^	2	NA	NA	NA	260
5Cl_2_HA=(Zr)			644							
NH_2_-MIL-88(Fe)	NH_2_, –COO^−^, Fe–O	Solvothermal	19.52	As^5+^	NA	4.8	97%	NA	NA	261
UiO-67-NH_2_ (1)	–NH_2_, –OH, –COO^−^	Modified solvothermal	750	_P_-ASA	80 mg L^−1^	4	161	Langmuir	PSO	262
UiO-67-NH_2_ (2)			465				178	Langmuir	PSO	
UiO-67			1871				278	Langmuir	PSO	
UiO-66-NH_2_	–NH_2_, –COO^−^, Zr-oxo	Solvothermal	855	As^5+^	100 mg L^−1^	7	161	NA	NA	263
MIL-101(OH)_3_	–OH	Direct solvothermal	2023	PAA	100 mg L^−1^	4–6	139	Langmuir	NA	264
				ASA	100 mg L^−1^		238	Langmuir	NA	
MIL-101(OH)			2119	PAA	100 mg L^−1^		84	Langmuir	NA	
				ASA	100 mg L^−1^		163	Langmuir	NA	
Pristine MIL-101			3557	PAA	100 mg L^−1^		57	Langmuir	NA	
				ASA	100 mg L^−1^		67	Langmuir	NA	
Am-UiO-66-NO_2_	–NH_2_, –OH, –COO^−^	Solvothermal	660	As^5+^	25 mg L^−1^	2	85	NA	NA	265
UiO-66-g-qP4VP/PAN (10%)	R_4_N^+^, –OH, –COO^−^, –C≡N	SI-ATRP method	3.3	As^5+^	200 mg L^−1^	8	162.17	Langmuir	PSO	266
UIO-66-TC-SH	–NO_2_, –OH, –COO^−^, SH	One pot method	457	As^3+^	200 mg L^−1^	7	193.46	Langmuir	PSO	267
Fe_3_O_4_@SiO_2_-MIL-53(Fe)	OH, –COO^−^, Fe–O–As	Hydrothermal	18.92	As^5+^	50 mg L^−1^	7	71.94	Langmuir	PSO	268
HP-UiO-66-40%	–OH, –COO^−^	Solvothermal, thermal	716	As^5+^	50 mg L^−1^	2–13	248.75	Langmuir	PSO	269
HP-UiO-66-30%			574	As^5+^			208.33			
NH_2_-MCM-44	–NH_2_, Si–OH	Sol gel	658	As^3+^	5 mg L^−1^	5.6	5.89	Langmuir	PSO	270
ZIF-67/S(iv)	SO_3_^2−^, Co^2+^	Precipitation	1771	As^3+^	50 mg L^−1^	9	185	Langmuir	PSO	271
				As^5+^			476			
UiO-66-SH	–SH, Zr–OH	Solvothermal	224	As^5+^	80 mg L^−1^	5	52.31	Langmuir	PSO	272
Fc-ZIF-67	Co^2+^, Fc	Solution mixing	NA	As^5+^	300 mg L^−1^	7	63.29	Langmuir	NA	273
NF/MIL-100(Fe)	–OH, –COOH, Ni^2+^	One pot hydrothermal	188.16	As^3+^	800 mg L^−1^	7	152.65	Langmuir	PSO	274
NF/MIL-100(Cr)			162.25			4	132.61			

## 5 Challenges and future directions

### 5.1 Challenges and limitations

Based on their tremendous surface area and tunable properties, although metal–organic frameworks (MOFs) have emerged as effective sorbents for arsenic removal, some challenges and limitations are hindering their widespread adoptions, which are as follows.

#### 5.1.1 Environmental stability and durability

The long-term applicability of MOFs is hindered by their long-term instability under various environmental conditions, especially in aqueous, acidic or basic environments. Hydrolytic stability is one of the most serious challenges in MOFs, occurs due to the hydrolytic cleavage of coordination bonds in the presence of water, which sometimes collapses the MOF structure and lowers its performance.^275,276^ While some Zr-based MOFs are usefully stable, they suffer from dissolution under basic conditions and are subjected to nucleophilic attack by hydroxides.^277^

Besides that, the thermal stability limits the applicability of the MOFs, since some MOFs may degrade above 250 °C.^278^ MOF stability can be improved further by the optimization of coordination environments, the introduction of rigid linkers, and the development of hydrophobic surface modifications, which limit water-mediated degradation.^279^ Another big concern is chemical solidity, especially under acidic and basic conditions. MOF structures may be disrupted by acidic or alkaline environments, which can lead to the leaching of metal ions and undesirable chemical reactions, thereby degrading their structural integrity and efficiency.^280,281^ To combat degradation, scientists have pursued coatings and hierarchical porous frameworks as potential solutions for stabilizing MOFs under extreme conditions.

#### 5.1.2 Regeneration and reusability

Practical implementations require MOFs to demonstrate consistent adsorption capacity throughout multiple cycles. However, many MOFs degrade after repeated adsorption–desorption cycles, resulting in a severe uptake capacity loss. For instance, certain MOFs such as BUT-155 and HKUST-1 frameworks exhibit poor hydrolytic stability.^282,283^ Designing a good adsorbate and easy regeneration with a long service life are very difficult to achieve at the same time, especially when high durability and ease of regeneration are required. Emerging regeneration approaches such as photocatalytic and solvent-based regeneration provide cleaner and more environmentally friendly alternatives for reactivating MOFs used in water treatment. These approaches not only restore MOF functionality but also minimize secondary waste generation.^284^

#### 5.1.3 Scalability and cost-effectiveness

Scaling up MOF production for commercial applications presents economic and technical challenges. Traditional synthesis methods such as solvothermal and hydrothermal methods are energy intensive and often expensive.^285,286^ Additionally, some MOFs incorporate precious metals, resulting in high cost, particularly when applied for catalytic purposes. To reduce production costs, researchers are exploring solvent-free synthesis methods such as mechanochemical synthesis, which aligns with green chemistry principles by minimizing solvent use and energy consumption.^287^ Furthermore, the development of biodegradable and recyclable MOFs could improve cost-effectiveness and environmental sustainability.^288^ Another promising approach involves flow chemistry and modular syntheses, which could enhance production efficiency and scalability while maintaining the product quality.^285^ Addressing these financial and technical barriers is essential for widespread MOF deployment in water treatment.

#### 5.1.4 Field testing and long-term studies

Despite their potential, MOFs encounter significant challenges such as long-term stability, environmental exposure and economic viability. The majority of MOFs degrade under high humidity, temperature fluctuation and chemical interaction. In particular, nickel-integrated MOFs exhibited unstable performance under high-temperature conditions, because the accumulation of nickel impurities diminishes the catalytic activity.^289^ Likewise, the use of some metal MOFs for arsenic adsorption is greatly reduced after several wet cycles, suggesting that moisture has a detrimental effect on their performance.^37^ This, coupled with the susceptibility of the MOFs to pH changes and ionic strength variation, complicated long-term use.^38^ For real-world placement, MOFs have to be validated under realistic conditions for their field studies and their long-term benefits compared with operational costs. While MOFs offer superior adsorption capabilities, the high intimal investment and maintenance costs often hinder their commercial viability.^290^ This drives the demand for economic assessments that compare various long-term benefits with relatively low initial investments, in order to evaluate the commercial potential of MOFs in various uses.

To evaluate the viability of MOFs in actual situations, field research is obligatory. It is noted that while laboratory outcomes for arsenic remediation are promising, the documentation of *in situ* remedy is still limited, indicating the need for further field testing.^291^ To address the possible remobilization of arsenic pollutants and understand the underlying mechanisms, it is essential to evaluate the long-term performance of chemisorption systems, including those based on iron oxide nanoparticles (IONPs) and MOFs.^292^ The need for continuous assessment is supported by studies calling for further research into long-term stability and environmental effects of MOFs across diverse environmental conditions.^293^

### 5.2 Future directions and research opportunities

#### 5.2.1 Designing sustainable and affordable MOFs

It is crucial to develop low-cost and environmentally friendly MOFs for widespread applications in arsenic removal. Key approaches include green synthesis methods, sustainable material selection, and energy-efficient production methods.^285^ Mechanochemical techniques offer a scalable and cost-effective alternative, which significantly reduced solvent use and energy consumption. Recent research suggests that biodegradable or recyclable MOFs such as calcium MIL-69, Cr-MIL-100, and Al-DA-MIL-53 can reduce the environmental impact.^294^ Zn-MOF/polyvinyl alcohol (PVA) composites have demonstrated high arsenic removal efficiency even after multiple sorption cycles.^51^ However, some challenges still remain in maintaining the structural integrity and functionality when modifying the MOF synthesis. To tackle this, the standardization of synthesis protocols and advanced characterization techniques are being explored to enhance reproducibility.^285^ Integrating sustainable practices in MOF manufacturing, together with machine learning-driven design optimization, holds the potential to enhance cost-efficiency, accelerate functionalization for arsenic capture, and promote wider applications.^58^ By mixing modular functionalities, rapid synthesis, alternative materials and life cycle assessments, researchers can develop MOFs that balance high-performance with sustainability, expanding their role in addressing environmental challenges.

#### 5.2.2 Innovative approaches for MOF regeneration

MOFs have the potential for long-term use in arsenic removal as they are critical for their long-term use that they can be regenerated efficiently. The conventional thermal regeneration approach can cause structural damage and high-energy consumption, which means that alternative new, environmentally friendly methods should be explored.^295^ Emerging non-thermal regeneration strategies such as solvent-based regeneration, UV irradiation, thermoresponsive materials, and photothermal techniques have shown promising potential to extend the lifespan of MOFs while minimizing the environmental impact.^296^ Some ionic liquids are more environmentally friendly, easier to regenerate and low cost compared to traditional high-temperature regeneration methods. Future needs to work on these in such a way to improve their scalability and efficiency with minimum energy consumption. As a result, the transformative shift towards energy conscious regeneration techniques will not only increase the performance of MOFs but also bolster the ability to implement them on a large scale for arsenic removal applications.

#### 5.2.3 Hybrid materials and functionalization

MOF hybridization and functionalization are excellent strategies to enhance their capacity, selectivity, and stability toward arsenic elimination.

• Hybridization of nanomaterials (*e.g.* CNTs and polymers) further improved the mechanical strength and arsenic adsorption effectiveness.^297^

• Post-synthetic modification with amino (NH_2_) or thiol (SH) groups enhanced selectivity for arsenic ions.^46^

• Harnessing electronic and redox modifications led to materials such as Fe-MWCNT composites with greater anodic oxidation properties, which are targeted towards arsenic-contaminated water treatment.^298^

Further advancements should be focused on synergistic approaches, where MOF functionalization coupled with catalytic and electrochemical methods to develop next-generation adsorbents. Therefore, combining these strategies together has the potential to achieve enhanced adsorption kinetics and stability of MOFs, rendering them more efficient as an efficient arsenic removal solution.

#### 5.2.4 Field testing and long-term studies

Even though there have been considerable laboratory improvements, MOFs must undergo stringent field experiments to confirm their durability of structure, chemical stability, and adsorption capacity under real-world scenarios.^299^

The key factors affecting MOF performance in natural settings include;

• Environmental variability (pH fluctuations, temperature changes, and water chemistry).

• Long-term structural stability (potential degradation and prolonged exposure).

• Adsorption–desorption cycling performance (sustainability of arsenic uptake capacity).

Prior research highlights the necessity for prolonged field trials to evaluate these variables. Some MOFs have been reported to lose their adsorption capability for arsenic after many cycles of wettability, which highlights the demand for stable materials that can withstand with changing environmental factors. Real-world performance evaluations must be integrated into MOF research, making the material selection, optimization, and commercialization process more controllable.^39,290,300^ Economic evaluations comparing long-term benefits with early investments in new solutions are needed to further establish the viability of broad adoption.

High-performance solutions for arsenic sequestration based on MOFs depend on their cost-effectiveness in synthesis, resource use in regeneration, blending with more commonly used materials and proper testing of the system on real and practical sources of sorbents. These considerations will enhance the performance, durability, and scalability of MOFs and set them up as promising candidates for practical water purification applications. Continued multidisciplinary research that interlaces materials science, environmental engineering, and sustainable chemistry will be crucial to unlocking the full potential of MOFs for heavy metal remediations.

## 6 Conclusions

MOFs are an emerging class of advanced materials used for the effective removal of arsenic from water systems. Their exceptional high surface area, tunable pore sizes, and varied chemical functionalities enable higher adsorption performance than the conventional materials. With their high specific surface area and suitable active species, MOFs can be specially designed to bind other ions including arsenic species, *via* electrostatic interactions, chelation, and ion exchange. These properties are crucial for the selective and targeted removal of As(iii) and As(v) species. The functionalization of MOFs with fixed groups (*e.g.*, amino, hydroxyl groups) improved their efficiency and selectivity for arsenic removal. Hybrid MOFs such as composites integrating polymers, magnetic nanoparticles, and graphene oxide provide additional advantages including ease of use and enhanced stability, making them incredibly effective arsenic adsorbents. Aqueous stability, large-scale production and long-lasting stability under diverse environmental conditions of pH and ionic strength are still important issues despite these developments.

To mitigate these challenges, new methods such as post-synthetic methods, microfluidic synthesis, and green chemistry methods have been developed. These approaches enhance the stability, scalability and environmental compatibility of MOFs. Moreover, new MOFs tailored to certain pollutants, and their inclusion in functional water treatment systems holds great promise in obtaining arsenic-free water. Ongoing efforts to tune MOF's structures and properties should lead to them becoming the primary materials for advanced water purification systems. MOFs are indeed pivotal in the fight to protect our ecosystems and deliver clean drinking water to billions around the world.

## Conflicts of interest

As the sole author, I am confirming that I have no known competing financial interests or personal relationships that could have appeared to influence the work reported in this paper.

## Data Availability

No primary research data, software, or code has been included in this review. No new data were generated or analysed during the preparation of this review.
