# Correction: Metal–organic frameworks (MOFs) for arsenic remediation: a brief overview of recent progress

**DOI:** 10.1039/d6ra90170k

**Published:** 2026-11-10

**Authors:** Easar Alam

**Affiliations:** a School of Environmental Engineering, Xuzhou University of Technology Xuzhou Jiangsu 221018 P.R. China easaralam@hotmail.com

## Abstract

Correction for ‘Metal–organic frameworks (MOFs) for arsenic remediation: a brief overview of recent progress’ by Easar Alam, *RSC Adv.*, 2025, **15**, 20281–20308, https://doi.org/10.1039/d5ra02420j.

In the original manuscript, the author regrets errors in the arsenic speciation terminology and surface area units. Throughout the manuscript, the arsenic species in aqueous systems are referred to as “As^3+^” and “As^5+^”. In water, however, arsenic should be described as arsenite As(iii) and arsenate As(v), rather than as free As^3+^ or As^5+^ ions. In addition, when discussing surface area throughout the manuscript, the surface area unit was incorrectly reported as m^3^ g^−1^. The correct unit is m^2^ g^−1^.

An independent expert has deemed that a Correction is appropriate in this case.

The results and conclusions of this paper are not affected by this correction.

The Royal Society of Chemistry apologises for these errors and any consequent inconvenience to authors and readers.

